# The glutathione pathway is required for biofilm formation in *Acinetobacter baumannii*

**DOI:** 10.1016/j.crmicr.2026.100562

**Published:** 2026-01-29

**Authors:** Jason M. Thomas, Pegah Mosharaf Ghahfarokhy, Samantha Gabrielle Perrotti Rivera, Clarissa J. Nobile, Mamta Rawat

**Affiliations:** a Department of Biology, California State University, Fresno, CA, USA; b Department of Molecular and Cell Biology, University of California Merced, Merced, CA, USA; c Quantitative and Systems Biology Graduate Program, University of California, Merced, CA, USA; d Health Sciences Research Institute, University of California, Merced, CA, USA

**Keywords:** Biofilms, Glutathione, Redox, *gshA*, *gshB*, ESKAPE pathogens

## Abstract

*Acinetobacter baumannii* is a Gram-negative nosocomial bacterium that is a member of the ESKAPE group of pathogens, notable for its virulence and intrinsic antibiotic resistance. It causes diverse infections, including respiratory and soft tissue disease, that are increasingly difficult to treat. Glutathione (GSH), the major intracellular redox buffer, is known in other bacteria to protect against stress and influence physiological processes such as biofilm formation. To investigate the roles of GSH in *A. baumannii*, we analyzed transposon mutant strains lacking *gshA* and *gshB* genes*,* which encode the enzymes that catalyze the two steps of GSH biosynthesis. Both mutant strains failed to produce GSH, exhibited impaired growth, and were hypersensitive to oxidative stress, nitrosative stress, toxins, and ferric chloride, compared to the wildtype strain. They also showed pronounced defects in biofilm formation and motility. Transcriptomic analysis of the *gshA* mutant strain relative to the wildtype strain revealed upregulation of genes involved in phenylacetate degradation and fimbrial biogenesis, while genes involved in iron and sulfur uptake and metabolism were downregulated. Moreover, mutant strains lacking GSH-dependent S-nitrosoglutathione reductases (GSNORs) displayed similar biofilm and nitrosative stress defects. Together, these findings demonstrate that GSH and GSNORs play central roles in stress resistance, biofilm development, and metabolic regulation in *A. baumannii*, highlighting their importance in the physiology and pathogenesis of this clinically relevant pathogen.

## Highlights

• Glutathione biosynthesis is required for GSH production in *A. baumannii*.
• Loss of GSH impairs stress resistance and motility.
• *gshA, gshB*, and *gsnoR* mutants are sensitive to nitrosative stress and impaired in biofilm formation.
• GSH deficiency alters expression of pili, siderophore, and metabolic genes.
• Exogenous GSH or GSH-containing media restores biofilm formation in mutants.

## Graphical abstract

**Figure:**
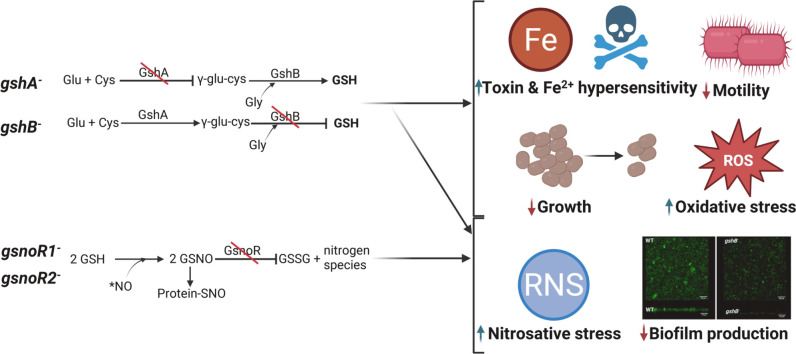

## Importance

*Acinetobacter baumannii* is an ESKAPE pathogen notorious for multidrug resistance and biofilm formation, traits that drive persistent hospital-acquired infections. However, the mechanisms enabling its survival under hostile conditions remain incompletely understood. Here, we show that glutathione (GSH), the primary redox buffer in *A. baumannii*, provides broad protection against environmental stresses, including oxidative and nitrosative damage, while also supporting key physiological processes such as motility and biofilm development. We further identify a critical interplay between GSH and nitric oxide (NO) in regulating biofilm formation. These findings establish GSH as a central determinant of *A. baumannii* stress tolerance and pathogenicity, offering new insights into pathways that may be targeted to combat this clinically important bacterium.

## Introduction

*Acinetobacter baumannii* is an aerobic, Gram-negative coccobacillus and a member of the ESKAPE group of nosocomial pathogens. It causes a wide range of infections, including respiratory tract infections, bacteremia, skin and soft tissue infections, urinary tract infections, and meningitis (Whiteway et al., 2022). Treatment is often difficult due to the organism’s intrinsic resistance to multiple antibiotics (Vázquez-López et al., 2020). In addition, *A. baumannii* readily forms biofilms on abiotic surfaces, such as catheters and ventilators, enabling persistence in diverse clinical environments (Sarshar et al., 2021). *A. baumannii* is known to produce a variety of virulence factors, including endotoxins, lipopolysaccharides, protective capsules, and phospholipases, as well as utilizes secretion and efflux systems that further enhance survival and pathogenicity within the host (Harding et al., 2018).

*A. baumannii* can survive in low-moisture environments, aided by capsular polysaccharides and under-acylated lipooligosaccharides in its outer membrane (Boll et al., 2015). Oxidative stress is a major consequence of desiccation, and the bacterium relies on enzymes such as the superoxide dismutase SodB, which converts superoxide radicals into hydrogen peroxide, and the catalases KatG and KatE, which detoxify hydrogen peroxide (Sun et al., 2016; Heindorf et al., 2014)*.* Accordingly, a *sodb* mutant strain shows increased sensitivity to oxidants, including hydrogen peroxide and paraquat, as well as to the antibiotic tetracycline (Heindorf et al., 2014). The *sodB* mutant strain also exhibits reduced motility in vitro and attenuated virulence in animal models (Heindorf et al., 2014). Similarly, *katG katE* double mutant strains display heightened sensitivity to hydrogen peroxide (Sun et al., 2016). Together, these findings underscore the importance of oxidative stress defense mechanisms in promoting *A. baumannii* survival, persistence, and pathogenicity under stressful environmental conditions.

Low molecular weight (LMW) thiols such as glutathione, (GSH, L-γ-glutamyl–L-cysteinyl– glycine) also contribute to protection against oxidative stress by maintaining cellular redox balance (Masip et al., 2006). GSH biosynthesis occurs in two sequential ATP-dependent steps: γ-glutamylcysteine synthetase (encoded by *gshA*) catalyzes the formation of γ-glutamylcysteine (γ-GC) from glutamate and cysteine, followed by GSH synthetase (encoded by *gshB*), which adds glycine to generate GSH (Meister, 1995). GSH is widespread among Gram-negative bacteria and present in some Gram-positive species (Fahey et al., 1978), whereas most Gram-positive bacteria rely on alternative thiols such as mycothiol (MSH) (Newton et al., 2008, Reyes et al., 2018) and bacillithiol (BSH) (Chandrangsu et al., 2018). Disruption of GSH, MSH, or BSH biosynthesis leads to diverse phenotypes, such as regulation of protein function via *S*-thiolation, cell cycle control, potassium homeostasis, deactivation of toxic compounds, and adaptation to environmental stresses, including oxidative, temperature, osmotic, and acid stress (Allocati et al., 2009, Arenas et al., 2010, Chardonnet et al., 2015, Ferguson et al., 1993, Hartl et al., 2020, Marcén et al., 2019, Riccillo et al., 2000, Helmann, 2011). More recently, LMW thiols have also been implicated in biofilm formation across multiple bacterial species, including *Pseudomonas aeruginosa* (Wongsaroj et al., 2018; Zhang et al., 2019; Van Laar et al., 2018), *Staphylococcus aureus* (Gulati et al., 2024), and *Mycobacterium smegmatis* (Vargas et al., 2016), a function thought to involve their capacity to mediate nitric oxide (NO) levels through a thiol-dependent nitrosothiol reductase. Interestingly, the connection between GSH, biofilm formation, and NO has been observed only with facultative anaerobic bacteria, and not with any aerobic bacteria, such as *A. baumannii*.

Although GSH is the major LMW thiol in *A. baumannii* (Walsh et al., 2020), its role in *A. baumannii* remains poorly defined. A *gshA* transposon mutant strain was recently identified in a genetic screen for genes required for surface-associated motility (Blaschke et al., 2021). In the related species *Acinetobacter baylyi*, a *gshA* transposon mutant strain was discovered to be sensitive to metronidazole, a prodrug derived from the nitroimidazole class of antimicrobial agents that generates DNA-damaging radicals upon activation (Gomez and Neyfakh, 2006). Interestingly, *gshA* is among the 30 genes induced under starvation in the well-studied *A. baylyi* strain ADP1 (Lostroh and Voyles, 2010). Building on these observations, we characterized *A. baumannii gshA* and *gshB* transposon mutant strains and found that, like other GSH-deficient strains, they are hypersensitive to toxins and defective in surface-associated motility. We further demonstrate that disruption of both of the genes encoding *S*-nitrosoglutathione reductases (GSNORs), which degrade S-nitrosoglutathione, impairs biofilm formation. Finally, transcriptomic analysis of the *gshA* mutant strain revealed altered expression of genes involved in diverse processes, including phenylacetate metabolism and iron uptake.

## Materials and methods

### Growth of strains

All bacterial strains and plasmids used in this study can be found in **Table 1S**. Transposon mutant strains in the *A. baumannii* AB5075 background were purchased from the University of Washington (Gallagher et al., 2015). Transposon insertion sites were confirmed by PCR amplification using the transposon-specific primer Pgro-172 and primers external to the mutated allele **(Table 1S)**. The transposon insertion site in the *gshA* transposon mutant strain was confirmed by comparing the *gshA* transcript levels in the mutant and wildtype strains **(Fig. S1)**. *A. baumannii* strains were cultured in Trypticase Soy Broth (TSB) unless otherwise specified. Antibiotics were added at the following concentrations: tetracycline (5 µg/mL) and apramycin (25 µg/mL).

Growth curves on liquid media were performed in triplicate in TSB. Overnight cultures of strains were diluted with fresh media to an OD_600_ = 0.2 and grown until cultures reached log phase (OD_600_ = 0.5). The cultures were then diluted to OD_600_ = 0.05 in 10 mL media in 50 mL Falcon tubes. OD_600_ was measured and colony forming units (CFUs) were counted at various time intervals.

### Genetic complementation of mutant strains

Complemented strains were constructed using the pMJG120 plasmid (a gift from Dr. M. Gebhardt at Boston Children’s Hospital (Gebhardt et al., 2015). Wildtype genes were cloned into pMJG120 in *Escherichia coli* DH5α using standard cloning techniques and gene specific primers flanking the open reading frame **(Table 1S)**. *E. coli* strains were cultured in Luria-Bertani broth (LB) at 37°C and 10 μg/mL apramycin was added for plasmid maintenance. Transformation of *A. baumannii* mutant strains with plasmids containing the wildtype genes was performed as previously described (Jacobs et al., 2014). The presence of the wildtype copy of the gene was verified by colony PCR using the same gene specific primer pair originally designed for the complementation cloning. PCR products were analyzed by agarose gel electrophoresis to confirm the presence and expected size of the insert.

### Measurement of LMW thiols

LMW thiols were measured by high-performance liquid chromatography (HPLC) analysis of fluorescent thiol adducts with monobromobimane as described previously (Johnson et al., 2009; Newton and Fahey, 1987; Fahey and Newton, 1987). Strains were grown to OD_600_ = 1.0 and harvested. Triplicate samples were analyzed from three different cultures.

### Sensitivity assays

For disk assays, overnight cultures were diluted to OD_600_ = 0.2 in TSB medium and incubated at 37°C until cultures reached OD_600_ = 0.5 and then 200 µL of culture was swabbed onto TSA plates. 7 mm disks (Fisher Scientific) with 10 µL of antibiotics of interest were placed onto the plates. The plates were incubated at 37°C for 14 h. Diameter zones of clearance and inhibition were measured (Rawat et al., 2007). Four independent experiments were performed in quadruplicate and statistical significance was performed using an unpaired two-tailed Student’s *t*-test.

Minimum inhibitory concentrations (MICs) for GSNO and sodium nitrite (NaNO_2_) were determined in ELISA plates by monitoring OD_600_. Cultures were grown for 12–16 h in TSB or acidified TSB (pH 5.0) media, diluted to a final OD_600_ of 0.01, and 50 µL was added to all wells, excluding blank (no cells) control wells. 50 µL of GSNO or NaNO_2_ of differing concentrations prepared in 100 µL TSB or acidified TSB (pH 5.0), respectively, was added to the wells. The plates were incubated with shaking at 37 °C for 24 h. The blank-subtracted OD_600_ values of each experimental well (three biological replicates per concentration) were divided by the blank-subtracted OD_600_ value of the control well to obtain normalized values. Reported data represent MICs for GSNO and NaNO_2_ with standard errors.

### Surface-associated motility

Surface-associated motility was performed as previously described with slight modifications (Blaschke et al., 2021). A single colony from a fresh TSA plate was plated on TSB containing 0.5% purified agar (Oxoid – ThermoFisher) with antibiotics where applicable. Plates were incubated for 16 h at 37°C. Diameter zones of spreading were measured. Each strain was assayed in quadruplicate at least three times. For each strain, data obtained from three independent experiments were averaged.

### Standard optical density biofilm assay

Cultures of *A. baumannii* were inoculated overnight into TSB or LB media with antibiotics when applicable at 37°C with shaking. Overnight cultures were diluted to an OD_600_ = 0.1 in TSB to obtain the initial inoculum, which was added into each well of a sterile 96-well polystyrene plate. Plates were placed in an ELMI shaker for 60 min at 37°C with shaking. Wells were then washed with PBS to remove non-adherent cells and 200 μL of fresh medium was added into each well. The plates were sealed with Breathe-Easy® membranes (Sigma–Aldrich) and incubated for an additional 48 h at 37°C with shaking. Following biofilm formation, media was aspirated from the wells, and the biofilms formed on the bottoms of each well was measured by taking OD_600_ readings using a BioTek Epoch 2 plate reader. An average of 24 reads per well were obtained and normalized by subtracting the OD_600_ reading of a blank well containing TSB or LB alone. The assay was performed using three independent biological replicates, each with two technical replicates. Statistical significance was determined using an unpaired Student’s *t*-test.

### Dry weight biofilm assay

Biofilms were formed as described above, with slight modifications to accommodate a 12-well plate format. Following incubation, wells were aspirated, and biofilms were vigorously disrupted in PBS by scraping and pipetting up and down. The suspended biofilms were collected and filtered through mixed cellulose ester membranes (MF-Millipore) using a vacuum filtration apparatus. Membranes were transferred to 6-well plates and incubated at 60°C for 30 min to dry. Biofilm biomass was quantified by weighing the dried membranes and subtracting the average weight of control membranes processed identically without cells. The assay was performed using three independent biological replicates, each with two technical replicates. Statistical significance was determined using an unpaired Student’s *t*-test.

### Confocal scanning laser microscopy (CSLM) biofilm assay

Autoclaved silicone squares were placed in each well of a 12-well plate using sterile forceps, 2 mL bovine serum was added to the wells and left overnight with shaking at 200 rpm, and the wells were washed with sterile PBS. After determining the cell density of overnight cultures of the bacterial strains, the cells were diluted to an OD_600_ = 0.1 in 2 mL of TSB with antibiotics (when applicable) and added to each well. The plates were incubated at 37°C for 60 min at 200 rpm in an ELMI incubator. Next, the media was aspirated from each well, and the wells were washed with 2 mL of sterile PBS. Fresh media was added, and the plates were incubated at 37°C for 48 h at 200 rpm. To fix biofilms, 80 μL of formaldehyde solution (37% in water) was added to each well for 20 min at 200 rpm in the ELMI incubator. After aspirating the media, 2 mL of H_2_O was added to each well, and the biofilms were subsequently stained with 5 μM Syto13 nucleic acid stain (Fisher Scientific) for 1 h at 37°C in the dark. Medium containing the stain was removed, 4 mL of PBS was added, and biofilms were visualized and imaged using a Zeiss LSM 880 confocal microscope with a water-dipping 40X objective lens. Z-Stacks were obtained at 652×652 pixels, imaging at intervals of 0.5 μm. Two biological replicates, each with three technical replicates and three random fields of view were analyzed, and representative images were selected. Biofilm top-view and side-view images were assembled using ImageJ. The .czi files were analyzed using the project stacks function in ImageJ to generate the side views of each stack.

Area of clumping was quantified using ImageJ. Raw microscopy images were first converted to 8-bit format and processed uniformly across the strains. For each strain, three independent fields of view were analyzed. Within each field of view, three aggregates were randomly selected, manually outlined using the selection tool, and the area of clumping (µm²) was measured using the calibrated scale. A total of nine aggregates per strain were quantified. The resulting measurements were used for statistical analysis, and comparisons between strains were performed by one-way ANOVA.

### RNA-seq analysis of wildtype and *gshA* transposon mutant strains

For RNA isolation, total RNA was extracted from bacteria grown to mid-logarithmic phase (OD_600_ = 0.4–0.6) in TSB using the RNeasy Mini Kit (Qiagen), according to the manufacturer’s instructions except that samples were lysed by bead-beating using a BeadBlaster 24 (Benchmark Scientific). To remove ribosomal RNAs, high-quality total RNA was processed using the Ribo-Zero Bacteria kit (Illumina). cDNA libraries were generated with rRNA depleted mRNA using the KAPA RNA Hyper Plus Kit (Roche), following the manufacturer’s instructions. Biological replicates were distributed across independent experimental batches to minimize potential batch effects in the RNA-seq analyses. Sequencing was conducted by QuickBiology using an Illumina HiSeq 4000 platform with 150 bp paired-end reads.

Reads were mapped to the *A. baumannii* reference genome (RefSeq GCF_000963825.1) using Rsubread version 2.4.0 (Liao et al., 2019). Gene expression was normalized with trimmed mean of M-values (TMM) using EdgeR, which was also employed for differential expression analysis and statistical testing (Robinson et al., 2010). Only mapped reads with counts of at least 10 per million and a *p*-value < 0.05 were included in downstream analyses. Functional categories and gene annotations for differentially expressed genes were assigned using BioCyc (Karp et al., 2019) and TIGRFAM (Haft et al., 2001).

### Quantitative real-time PCR (qPCR) validation of RNA-seq results

qPCR was performed using the same RNA used for RNA-Seq. Reverse transcription of 1 µg total RNA was performed using M-MLV Reverse Transcriptase (Promega) and random primers (Promega) to yield cDNA. A second reaction that did not contain M-MLV Reverse Transcriptase served as a negative control. To confirm cDNA synthesis and that no gDNA was present, PCR using primers for the housekeeping genes was performed on the samples processed with reverse transcriptase. qPCR was performed using 50 ng of cDNA or RNA (as a negative control), 0.2 pmol of each primer, and 10 µL of Absolute qPCR SYBR Green mix (Thermo Scientific), with the following PCR program: 50°C for 10 min, 95°C for 15 min, followed by 40 cycles of 95°C for 30 s, 58–60°C for 30 s, 72°C for 1 min, with read occurring at 74°C for 30 s, and then a melt curve of 95°C for 30 s, 60°C for 30 s, and up to 95°C over 20 min. Relative gene expression was calculated using the -2^∆∆CT^ method (∆C_t_ = C_t sample_ – C_t control_) and reported as fold change (Schmittgen and Livak, 2008) after normalization to housekeeping genes, 16S (Sun et al., 2016) and *gyrB* (Rumbo-Feal et al., 2013). Primers used for qPCR analyses are listed in **Table S1.**

### Data availability

RNA-seq data has been deposited in NCBI's Gene Expression Omnibus (GEO) under accession number GSE192957.

## Results

### The *gshA* and *gshB* transposon mutant strains lack detectable glutathione

Transposon mutant strains in *gshA* (AB00324) and *gshB* (AB00412) were confirmed by PCR. The *gshA* mutant strain carried an insertion 369 bp from the 5′ end of ABUW_0114, which is located in an operon with *dsbB* (ABUW_0115), a gene involved in periplasmic disulfide bond formation. We note that *dsbB* appears to be transcribed normally in both the *gshA* mutant and WT strains as determined by our RNA-seq, and that *dsbB* is not differentially expressed between the *gshA* mutant and the WT strain, indicating that *dsbB* expression levels are not affected by the mutation in *gshA*. The *gshB* mutant strain carried an insertion 467 bp from the 5′ end of ABUW_0145, a monocistronic locus. Thiol analysis was performed on wildtype (AB5075), mutant, and complemented strains grown in TSB, a medium with undetectable background GSH (Fahey et al., 1978; Newton et al., 1993).

No GSH was detected in the *gshA* mutant strain and complementation restored GSH to partial levels (2.37 ± 0.07 µmol/g dry weight). Similarly, the *gshB* mutant strain lacked GSH but accumulated elevated levels of the substrate γ-glutamylcysteine (γ-GC; 8.50 ± 0.33 µmol/g dry weight). Complementation restored GSH production (5.0 ± 0.0 µmol/g dry weight) and reduced γ-GC levels (0.9 ± 0.0 µmol/g dry weight), although GSH remained only partially restored. Interestingly, the *gshB* complemented strain accumulated approximately twofold more GSH than the *gshA* complemented strain. Cysteine was detected only in the *gshB* mutant strain and its complemented derivative (Table 1). These results validate the annotation of ABUW_0114 and ABUW_0145 as *gshA* and *gshB*, respectively, and demonstrate that both genes are essential for GSH biosynthesis in *A. baumannii* (Whiteway et al., 2022).

**Table 1:** **Thiol levels in *A. baumannii* strains (GSH-glutathione, γ-GC- γ-glutamylcysteine, CSH-cysteine)**. Means of quadruplicate replicates ± standard deviation are reported. Based on previously validated methods, the HPLC detection limits for the analytes measured in this study are <0.05 (Johnson et al., 2009; Newton and Fahey, 1987; Fahey and Newton, 1987). Student *t*-tests were used to evaluate significance (* ***P*** ≤ 0.05, ** *P* ≤ 0.005, ***P ≤ 0.0005 as compared to WT (AB5075-UW)).

**Strain**	**Thiol concentration (µmole/g dry wt)**		
	GSH	γ-GC	CSH
WT (AB5075-UW)	6.9 ± 0.5	<0.05	<0.05
WTev (AB5075-UW::pMJG120)	7.4 ± 1.5	<0.05	<0.05
*gshA^−^* (AB00324)	<0.05***	<0.05	<0.05
gshA-C (AB00324-JTgshA)	2.4 ± 0.1***	<0.05	<0.05
*gshB*^−^ (AB00412)	<0.05 ***	8.5 ± 0.3*	0.1 ± 0.0**
gshB-C (AB00412-JTgshB)	5.0 ± 0.0*	0.9 ± 0.0***	0.1 ± 0.0***

### A lack of GSH impairs growth in liquid tryptic soy broth media

**Fig. 1:**
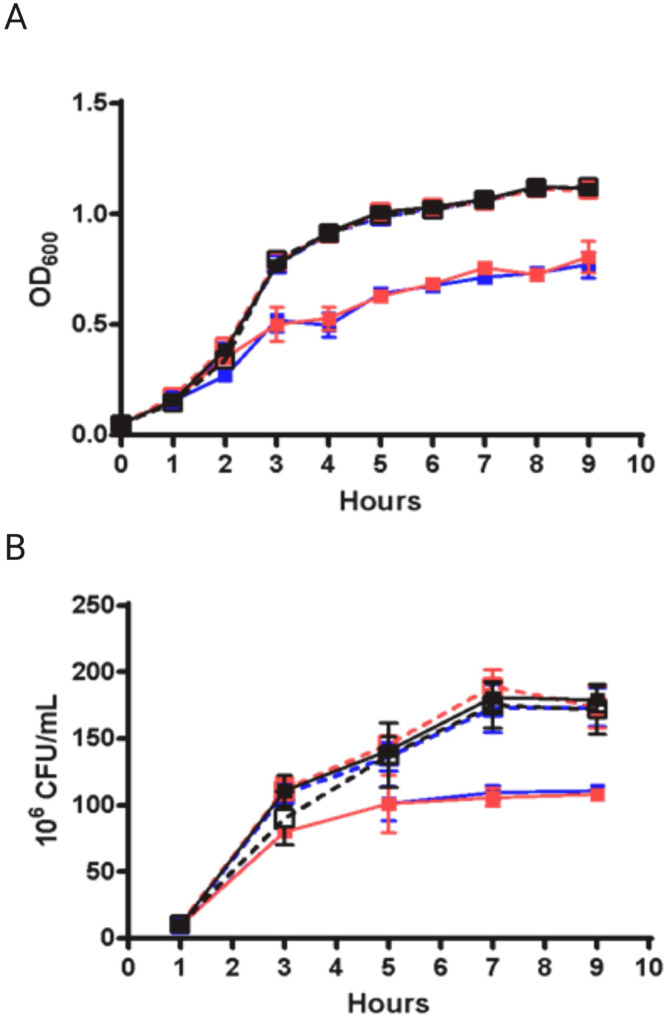
**Growth rates of *A. baumannii gsh* mutant strains in liquid tryptic soy broth media.** Strains were grown in triplicate in 10 mL media in 50 mL conical tubes. Logarithmically growing cultures were diluted to 0.05 OD_600_ in TSB and incubated at 37 °C with shaking. Aliquots were taken at specific timepoints and (a) OD_600_ and (b) CFUs were determined; wildtype (black solid line, closed square), WTev (black dashed line, open square), *gshA^−^ (*red solid line, closed square), gshA-C (red dashed line, open square), *gshB^−^* (blue solid line, closed square), gshB-C (blue dashed line, open square). Error bars indicate standard deviation calculated from three independent biological replicates at each timepoint.

Both *gshA* and *gshB* mutant strains exhibited reduced growth in TSB, reaching stationary phase at lower optical densities than the wildtype and complemented strains (Fig. 1A). Colony forming units (CFUs) confirmed this growth defect (Fig. 1B). Complementation with the respective wildtype genes fully restored growth (Fig. 1A and B), demonstrating that the phenotype was specifically due to loss of GSH biosynthesis.

### Disruption of GSH biosynthesis alters susceptibility to oxidants, metals, and toxins

Because bacterial mutants lacking LMW thiols often show altered stress responses (Van Laar et al., 2018; Fang and Dos Santos, 2015; Helbig et al., 2008; Rawat et al., 2007; Rajkarnikar et al., 2013; Williams et al., 2018; Potter et al., 2012), we compared the sensitivity of *A. baumannii* AB5075 wildtype, *gshA* and *gshB* mutant strains, and their respective complemented strains to a range of stressors. The *gshA* mutant strain was hypersensitive to hydrogen peroxide (H_2_O_2_), paraquat, and cumene hydroperoxide (CHP), whereas the *gshB* mutant strain was sensitive to paraquat and CHP but not H_2_O_2_ (Table 2). Neither mutant strain was sensitive to the thiol oxidant diamide (Table 2). The relative resistance of the *gshB* mutant strain to H₂O₂ may reflect compensation by elevated γ-glutamylcysteine (γ-GC) levels, which can act as an alternative reducing agent in peroxide detoxification.

**Table 2:** **Zones of clearance for *A. baumannii* strains with oxidants** o**n tryptic soy agar plates.** Means of quadruplicate replicates ± standard deviations are reported. Student *t*-tests were used to evaluate significance (* ***P*** < 0.05, ** *P* ≤ 0.005, ***P ≤ 0.0005 as compared to wildtype).

Reagents (µmol)	WT	*gshA^−^*	gshA-C	*gshB^−^*	gshB-C	WTev
Paraquat (1.0)	12.8 ± 0.5	19.3 ± 1.0***	15.0 ± 1.4*	14.5 ± 0.6***	12.0 ± 0.8	12.3 ± 0.5
H_2_O_2_ (0.5)	12.5 ± 0.6	14.8 ± 0.5***	13.0 ± 0.0	12.8 ± 0.5	12.0 ± 0.0	12.0 ± 0.8
CHP (1.0)	16.5 ± 1.0	20.0 ± 0.8***	17.0 ± 0.8	21.0 ± 0.0***	16.3 ± 0.5	17.0 ± 0.8
Diamide (2.5)	18.9 ± 0.9	18.8 ± 0.5	18.0 ± 0.8	19.3 ± 0.5	19.0 ± 0.0	18.8 ± 1.0

When tested against metals, both mutant strains displayed increased sensitivity only to ferric ions (Table 3). We also tested whether the wildtype (AB5075), *gshA,* and *gshB* mutant strains were sensitive to ampicillin since strain specific differences in sensitivity to ampicillin have been reported. Like the ATCC 17978 *gshA*::Km mutant strain but unlike the 29D2 *gshA*::Km mutant strain, our *gshA* mutant strain was not sensitive to ampicillin (Blaschke et al., 2021) (Table 4). When the canonical glutathione *S*-transferase (GST) substrate, 1‑chloro-2,4-dinitrobenzene (CDNB), was tested, the *gshA* mutant strain was sensitive to this toxin, but the *gshB* mutant strain was not (Table 4). Finally, both mutant strains were sensitive to methylglyoxal, a detrimental metabolic byproduct that is predominantly detoxified by its conjugation with GSH (Table 4). In all cases, complementation with the corresponding wildtype gene restored resistance to wildtype levels.

**Table 3:** **Zones of clearance for *A. baumannii* strains with metals** o**n tryptic soy agar plates.** Means of quadruplicate replicates ± standard deviations are reported. Student *t*-tests were used to evaluate significance (* ***P*** < 0.05, ** *P* ≤ 0.005, ***P ≤ 0.0005 as compared to wildtype). NC= no clearing.

Reagents (µmol)	WT	*gshA^−^*	gshA-C	*gshB^−^*	gshB-C	WTev
Na_2_SeO_3_ (10)	NC	NC	NC	NC	NC	NC
CdSO_4_ (1.25)	15.0 ± 0.0	15.3 ± 0.5	15.0 ± 0.8	14.5 ± 0.6	14.8 ± 1.5	13.8 ± 1.0
FeSO_4_ (40)	13.5 ± 0.6	14.0 ± 0.8	13.5 ± 1.0	13.3 ± 1.0	14.0 ± 0.8	12.0 ± 1.2
FeCl_3_ (30)	12.3 ± 1.0	15.3 ± 0.5***	12.8 ± 0.5	15.0 ± 0.0*	12.5 ± 0.6	12.8 ± 0.5
CuSO_4_+Ac (5.0)	10.0 ± 0.8	10.3 ± 0.5	9.5 ± 0.6	9.8 ± 0.5	10.0 ± 0.0	9.5 ± 0.6
CuSO_4_ (40)	20.5 ± 0.6	21.3 ± 1.3	19.8 ± 1.0	21.8 ± 1.0	22.3 ± 1.3	21.5 ± 1.0
K₂CrO₄ (0.2)	14.5 ± 0.6	12.8 ± 1	12.5 ± 1.3	13.5 ± 0.6	14.5 ± 1.7	14.5 ± 1.7
K_2_Cr_2_O_7_ (0.2)	22.3 ± 0.5	22.0 ± 0.0	23.3 ± 1	22.5 ± 0.6	21.0 ± 1.4	23.0 ± 0.8

**Table 4:** **Zones of clearance for *A. baumannii* strains to toxins and antibiotics grown on tryptic soy agar plates.** Means of quadruplicate replicates ± standard deviations are reported. Student *t*-tests were used to evaluate significance (* ***P*** < 0.05, ** *P* ≤ 0.005, ***P ≤ 0.0005 as compared to wildtype).

Reagents (µmol)	WT	*gshA^−^*	gshA-C	*gshB^−^*	gshB-C	WTev
CDNB (1.0)	18.5 ± 0.6	23.5 ± 0.6***	20 ± 0.8*	18.3 ± 1.0	18.8 ± 1.0	18.5 ± 1.3
Ampicillin (2.5)	11.8 ± 0.5	11.8 ± 0.5	11.8 ± 1.0	11.5 ± 0.6	10.5 ± 0.6	12.3 ± 1.0
MG (5.0)	18.8 ± 0.5	22.3 ± 1.0***	19.8 ± 0.5*	20.8 ± 0.5***	19.0 ± 0.8	18.8 ± 1.0

### Disruption of GSH biosynthesis and *gsnoR* genes increases susceptibility to nitrosative stress

We assessed the sensitivity of *A. baumannii* strains lacking GSH biosynthesis to nitrosative stress using *S*-nitrosoglutathione (GSNO) and acidified sodium nitrite (NaNO_2_), building on previous findings that mutant strains deficient in LMW thiols are more susceptible to nitrosative stress (Vargas et al., 2016; Gulati et al., 2024). The MICs for both GSNO and NaNO_2_ were fourfold lower for the *gshA* and *gshB* mutant strains (2.5 mM) compared to the wildtype strain (10 mM,Table 5).

**Table 5:** **Minimum inhibitory concentrations of *A. baumannii* strains grown** i**n liquid tryptic soy broth (+GSNO) or acidic tryptic soy broth (+NaNO_2_).** MIC values (mM) are presented as mean ± standard error of the mean from three biological replicates.

Strain	MIC (mM)	
	GSNO	NaNO_2_
WT	10.0 ± 0.0	10.0 ± 0.0
*gshA^−^*	2.5 ± 0.0	2.5 ± 0.0
*gshB^−^*	2.5 ± 0.0	2.5 ± 0.0
*gsnoR*1*^−^*	2.5 ± 0.0	2.5 ± 0.0
*gsnoR*2*^−^*	2.5 ± 0.0	5.0 ± 0.0
gshA-C	10.0 ± 0.0	10.0 ± 0.0
gshB-C	10.0 ± 0.0	10.0 ± 0.0
gsnoR1-C	10.0 ± 0.0	10.0 ± 0.0
gsnoR2-C	10.0 ± 0.0	10.0 ± 0.0

We also evaluated strains disrupted in *S*-nitrosothiol reductases/formaldehyde dehydrogenases, which utilize LMW thiols as cofactors to reduce *S*-nitrosothiols via NADH/NADPH or oxidize aldehydes while reducing NAD+/NADP+ (Chen et al., 2013; Liu et al., 2001). *A. baumannii* has two putative *S*-nitrosothiol reductases, *gsnoR1* (ABUW_2057W) and *gsnoR2* (ABUW_2594), which share sequence similarity with confirmed GSNORs and possess known formaldehyde dehydrogenase activity (**Fig. S1**) (Echenique et al., 2001).

The *gsnoR1* mutant strain exhibited MICs identical to the GSH mutant strains (2.5 mM), suggesting its activity depends on GSH. The *gsnoR2* mutant strain displayed slightly higher MICs (2.5–5.0 mM) but remained more sensitive than the wildtype (10 mM,Table 5). These results indicate that both *gsnoR* genes contribute to nitrosative stress resistance, with *gsnoR1* playing a dominant role that is dependent on GSH.

### Surface-associated motility is impaired in *gshA* and *gshB* mutant strains

**Fig. 2:**
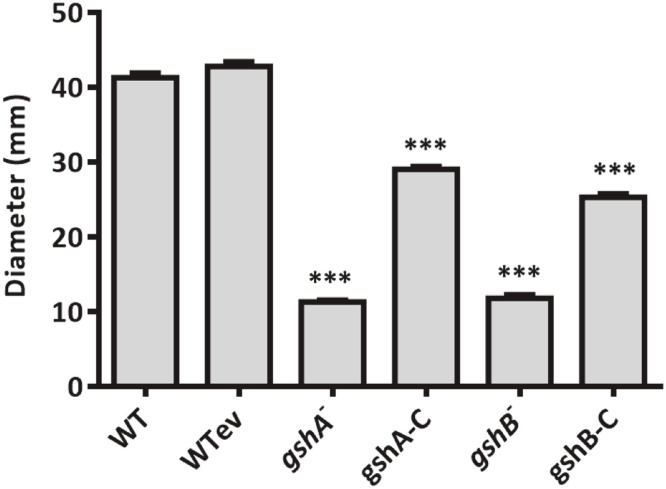
**Surface-associated motility of the *gsh* mutant strains**. Surface-associated motility was performed as previously described in (Blaschke et al., 2021), with slight modifications. A single bacterial colony from a fresh tryptic soy agar plate was replated on a tryptic soy agar plate. Plates were supplemented with antibiotics when applicable. Plates were incubated for 16 h at 37 °C. The diameter of the spreading zone was measured. Each strain was assayed in quadruplicate and data are presented as mean ± standard error of the mean. Student's *t*-tests were performed to determine significance; *** *P* ≤ 0.001.

Consistent with previous observations in *A. baumannii gshA* mutant strains (Blaschke et al., 2021), we assessed surface-associated motility in our *gshA* and *gshB* transposon mutant strains and their complemented strains. Both mutants exhibited markedly reduced motility, which was partially restored upon complementation with the corresponding wildtype gene (Fig. 2).

### Disruption of GSH and *gsnoR* genes reduces biofilm formation

Previous studies have shown that loss of LMW thiols impairs biofilm formation in other bacteria, including *Pseudomonas aeruginosa* (*gshA* mutant) (Van Laar et al., 2018, Zhang et al., 2019a), *Staphylococcus aureus* (*bshC* mutant) (Gulati et al., 2024), and *Mycobacterium smegmatis* (*mshC* mutant) (Vargas et al., 2016), and that *S*-nitrosothiol reductase mutant strains also display biofilm defects in *S. aureus, M. smegmatis*, and *Neisseria meningitidis* (Gulati et al., 2024; Vargas et al., 2016; Chen et al., 2013).

**Fig. 3:**
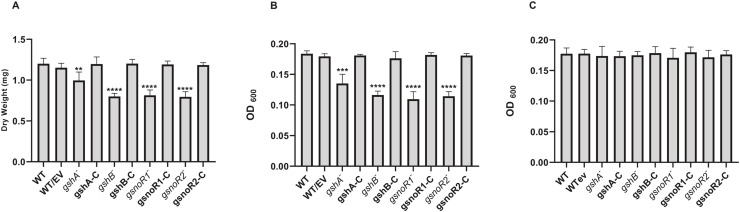
**Biofilm formation in *A. baumannii gsh* and *gsnoR* mutant strains.** (a) Biofilm dry weight assay in tryptic soy broth, (b) biofilm optical density assay in tryptic soy broth, (c) biofilm optical density assay in LB. *A. baumannii gshA, gshB, gsnoR1*, and *gsnoR2* mutant, complemented, and isogenic wildtype strains were examined. The assay was performed with three independent biological replicates, each with two technical replicates. Data are presented as mean ± standard error of the mean. Statistical significance was determined using an unpaired Student’s *t*-test. *P*-values are indicated as * = 0.05, ** = 0.01, *** = 0.001, **** = 0.0001.

**Fig. 4:**
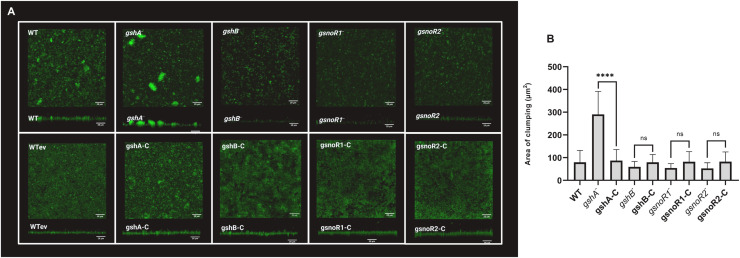
**Confocal scanning laser microscopy (CSLM) analysis of biofilms.** (a) Top-views (upper) and side-views (lower) CSLM images of representative biofilms formed by *A. baumannii* strains: WT, WTev, *gshA^−^*, gshA-C, *gshB^−^*, gshB-C, *gsnoR1^−^*, gsnoR1-C, *gsnoR2^−^*, and gsnoR2-C. Biofilms were grown in tryptic soy broth in 12-well plates, stained with Syto13 nucleic acid stain, and imaged using a Zeiss LSM 880 confocal microscope using a 488/561 nm diode laser. Z-stacks were acquired at 652 × 652 pixels with 0.5 μm intervals using a 40× water-dipping objective. Images were processed in ImageJ using the "Project Stacks" function to generate top and side views. Scale bars = 25 μm. (b) The area of clumping (µm²) of the strains. Aggregate areas were quantified from the CSLM images by selecting three clumped aggregates from three independent fields of view per strain. The *gshA^−^*mutant formed significantly larger aggregates compared to the WT and its complemented strain. Data plotted represents mean aggregate area, and error bars indicate standard deviation. Statistical analysis comparing each mutant strain to its respective complemented strain is shown and was performed by one-way ANOVA. ns, not significant; **** *P* < 0.0001.

We measured biofilm formation in *A. baumannii* transposon mutant strains lacking GSH biosynthesis or *gsnoR* genes. All mutant strains exhibited reduced biofilm formation, as assessed by standard biofilm dry weight (Fig. 3A) and biofilm optical density assays (Fig. 3B). Specifically, the *gshB, gsnoR1*, and *gsnoR2* mutant strains formed ∼30% less biofilm than the wildtype, while the *gshA* mutant formed ∼20% less biofilm than the wildtype. Complementation with the respective wildtype genes restored biofilm formation to wildtype levels. Notably, when grown in LB, which contains trace GSH, no differences in biofilm formation were observed among the strains (Fig. 3C). To investigate morphological differences between the strains, we used confocal scanning laser microscopy (CSLM) to visualize the biofilms, which revealed pronounced cell clumping in *gshA* mutant biofilms compared with the other strains (Fig. 4).

### Loss of GSH results in differential gene expression in *A. baumannii*

To investigate the consequences of GSH depletion, we performed RNA-seq on *A. baumannii* AB5075 wildtype and *gshA* transposon mutant strains. The disruption of *gshA* in the mutant was confirmed (**Fig. S2**). Sequencing yielded 8.6–9.6 million reads per sample, with 89–91% mapping to the reference genome (**Table S2**). Multidimensional scaling (MDS) plots indicated distinct transcriptomic profiles between the wildtype and mutant strains (**Fig. S3**). Comparative analysis revealed 39 genes upregulated (log₂FC >1.0) and 126 genes downregulated (log₂FC < -1.0) in the mutant versus the wildtype strains (**Tables S3–S4**), with only 8 genes exhibiting log₂FC >2.0 and 9 genes exhibiting log₂FC < -2.0 (Table 6). A subset of genes was validated by qPCR, confirming the RNA-seq results (**Table S5**).

**Table 6:** Highly upregulated and downregulated genes identified in the *A. baumannii gshA* mutant strain compared to the wildtype strain.

Upregulated				
Annotation	Gene	Description	Log_2_FC	*P*-value
ABUW_RS07200	ABUW_1477	*gltS* sodium/glutamate symporter	3.2	1.22E-07
ABUW_RS07220	ABUW_1481	SDR family oxidoreductase	2.4	3.22E-05
ABUW_RS07225	ABUW_1482	SRPBCC family protein	2.3	4.20E-05
ABUW_RS07255	ABUW_1488	fimbrial major subunit CsuA/B family protein	2.3	2.24E-03
ABUW_RS07280	ABUW_1493	hypothetical protein	2.1	3.83E-02
ABUW_RS07205	ABUW_1478	cyclase family protein	2.1	3.97E-05
ABUW_RS07260	ABUW_1489	*csuB*	2.0	3.77E-05
ABUW_RS07215	ABUW_1480	aldo/keto reductase	2.0	3.22E-05

Downregulated				
Annotation	Gene	Description	Log_2_FC	*P*-value
ABUW_RS10185	ABUW_2097	CoA transferase subunit B,		
3-oxoadipate CoA-succinyl transferase beta subunit	-3.3	3.22E-05		
ABUW_RS10180	ABUW_2096	CoA transferase subunit A	-3.2	8.80E-07
ABUW_RS11265	ABUW_2316	EamA family transporter	-2.6	3.77E-05
ABUW_RS07680	ABUW_1572	3-hydroxyacyl-CoA dehydrogenase	-2.6	4.22E-05
ABUW_RS07685	ABUW_1573	butyryl-CoA dehydrogenase	-2.3	4.72E-05
ABUW_RS11940	ABUW_2454	3-methylglutaconyl-CoA hydratase	-2.1	5.38E-05
ABUW_RS00205	ABUW_0042	2,5-didehydrogluconate reductase DkgB	-2.1	2.92E-04
ABUW_RS18510	ABUW_3803	IS5-like element transposase	-2.1	4.20E-02
ABUW_RS13010	ABUW_2679	DUF4142 domain-containing protein	-2.1	1.66E-03

Several key physiological processes were affected in the *gshA* mutant strain. The most upregulated gene was *gltS* (ABUW_1477; log₂FC 3.23), encoding a sodium/glutamate symporter. Glutamate serves as a major metabolic hub, contributing to nitrogen assimilation, nucleotide and amino acid biosynthesis, secondary metabolite production, and desiccation tolerance (König et al., 2020). Upregulation of *gltS* and the potassium-transporting ATPase subunits *kdpA* and *kdpB* (log₂FC 1.7 and 1.4) suggests altered sodium-potassium homeostasis, which may be further exacerbated by impaired GSH-dependent activation of KefB/KefC potassium efflux systems (Ferguson et al., 1993). Downregulation of an *eamA* family transporter (log₂FC -2.6) indicates broader impairment of small molecule transport.

Genes involved in fimbrial biosynthesis were largely upregulated, including *CsuA/B* (log₂FC 2.3) and Csu fimbrial biogenesis genes (*CsuB–E*, log₂FC 1.5–2.0,Table 6, **Fig. S4**). These structures mediate attachment to abiotic surfaces and microcolony formation (Pakharukova et al., 2018; Ahmad et al., 2023). A neighboring hypothetical protein, ABUW_1493, was also upregulated, suggesting co-regulation. In contrast, a periplasmic fimbrial chaperone (ABUW_2311) was downregulated, consistent with reduced biofilm adherence. Other biofilm-associated systems, including Type I SS, Bap, RTX, PNAG, and Pap pili homologs, were not differentially expressed (Upmanyu et al., 2022).

Notably, eight of eleven genes in the phenylacetic acid (PAA) catabolism pathway were upregulated (log₂FC >1.0), potentially impacting TCA cycle intermediates and neutrophil chemotaxis via excreted PAA **(Table S3, Fig. S5)** (Bhuiyan et al., 2016, Hooppaw et al., 2022). Conversely, genes involved in isoleucine and leucine catabolism, including ABUW_1572–1573 and the ABUW_2452–2456 operon, were downregulated, reflecting possible auxotrophy as observed in other LMW thiol mutants **(Table S4**) (Fang and Dos Santos, 2015). Genes encoding Fe-S cluster proteins, such as ABUW_2436 (*KatE*) and ABUW_2437, were also downregulated, linking GSH depletion to disrupted Fe-S-dependent metabolism **(Table S4**) (Mühlenhoff et al., 2020).

Iron homeostasis was perturbed: preacinetobactin-acinetobactin biosynthesis genes (*basEFG, basB*) and the transporter *bauA* were downregulated, as were baumannoferrin biosynthesis genes (*bfnA/B*), whereas fimsbactin genes were unchanged (**Table S4, Fig. S6**). Other metal homeostasis genes, including a P-type ATPase (ABUW_3325) and a multicopper oxidase (ABUW_3321), were downregulated. Upregulated genes under zinc stress (*ABUW_1480–1481*, log₂FC 2.0–2.4) may relate to impaired motility (Alquethamy et al., 2019).

Sulfur metabolism was broadly affected (Pokhrel et al., 2023; Tomlinson et al., 2022): operons for sulfate (*cysT/W*), taurine, and sulfonate metabolism were downregulated (**Fig. S7**), along with *sbp* (ABUW_0280), *SfnB* (ABUW_2336), and cysteine desulfurase (*ABUW_2417*). Methionine biosynthesis (*metE*, ABUW_3197) was also reduced, suggesting compromised sulfur utilization **(Table S4).**

Other metabolism related genes were downregulated, including FADH-dependent acyl-CoA dehydrogenases (*ABUW_3772, ABUW_2452*), NAD(P)/FAD-dependent oxidoreductases (*ABUW_2767, ABUW_2337*), and *dkgB* (log₂FC -2.1), implicating disrupted electron transport, redox balance, and glucose utilization **(Table S4)**.

Finally, several transcriptional regulators were differentially expressed. Upregulated regulators included a LysR family TR (ABUW_1479; log₂FC 1.5), likely redox-responsive, whereas TetR/AcrR family TRs (ABUW_1159, ABUW_2451; log₂FC -1.4, -1.4) were downregulated. These regulators influence metabolism, efflux, biofilm formation, and virulence **(Table S3, Table S4)** (Baugh et al., 2023, Cuthbertson and Nodwell, 2013).

Overall, the RNA-seq data indicate that GSH depletion broadly perturbs ion homeostasis, metabolism, biofilm-related gene expression, and stress response pathways, providing a mechanistic basis for the phenotypes observed in the *gshA* mutant.

## Discussion

In this study, we demonstrate that *gshA* and *gshB* are responsible for GSH production in *A. baumannii*. Mutant strains disrupted in either of the genes, grown on TSB, which contains undetectable GSH, did not produce GSH (Table 1). While *A. baumannii* possesses putative GSH/GSSG import systems (Opp and Gsi; ABUW_2836, ABUW_2220–2226, ABUW_2837–2839) (Knoke et al., 2025), allowing uptake of exogenous GSH from media such as LB (Helbig et al., 2008), endogenous biosynthesis is clearly critical under conditions lacking GSH.

The *gshA* and *gshB* mutant strains exhibited increased sensitivity to oxidants, nitrosative stress, ferric ions, and toxins, consistent with observations in other bacterial species (Table 2, Table 3, Table 4, Table 5). Interestingly, the *gshB* mutant strain was resistant to H₂O₂, likely due to the accumulation of γ-glutamylcysteine (γ-GC), which may partially compensate for GSH in peroxide detoxification. *A. baumannii* also encodes putative GSH peroxidases (ABUW_2355, ABUW_3729) capable of reducing H₂O₂ using GSH or γ-GC, although the genes encoding these enzymes, along with other ROI-detoxifying genes, were downregulated in the *gshA* mutant strain (**Table S6**).

GSH deficiency also perturbed iron homeostasis. GSH can chelate metals, including iron (Hider and Kong, 2011), and our RNA-seq data revealed downregulation of genes involved in siderophore synthesis (*basEFG, basB*) and transport (*bauA*), as well as baumannoferrin biosynthesis (*bfnA/B*,**Fig. S6**). Coupled with GSH’s ability to reduce ferric ions (Park and Imlay, 2003), its absence likely increases susceptibility to ferric iron, consistent with our phenotypic data.

GSH also influences motility. Mutant strains lacking GSH, such as the *P. aeruginosa gshA* mutant strain, exhibit decreased swarming, swimming, and twitching motility (Zhang et al., 2019; Van Laar et al., 2018; Wongsaroj et al., 2018). Both *gshA* and *gshB* mutant strains displayed impaired surface-associated motility (Fig. 2), consistent with previous reports (Blaschke et al., 2021). Similar motility defects occur in *A. baumannii sod* mutant strains (Heindorf et al., 2014) and in *Salmonella* strains lacking superoxide dismutase [44], suggesting that ROS detoxification may modulate signaling pathways controlling motility. Upregulation of Csu pili genes in the *gshA* mutant strain (**Fig. S4**) could further suppress surface-associated motility, as Csu pili have been reported to inhibit motility while enhancing colonization in vivo (Ahmad et al., 2023).

GSH and *S*-nitrosothiol reductases also influence biofilm formation. Despite Csu pili upregulation—which would be expected to promote biofilm attachment—*gshA, gshB*, and *gsnoR* mutant strains exhibited impaired biofilm formation in TSB (Fig. 3A–B). In LB, containing trace GSH, biofilm formation was restored (Fig. 3C), highlighting the importance of intracellular GSH levels. Among the *gsnoR* mutants, *gsnoR1* exhibited higher sensitivity to nitrosative stress than *gsnoR2*, suggesting partial functional redundancy or compensatory activity by other enzymes. Downregulation of *gsnoR2* in the *gshA* mutant implies that LMW thiols may regulate GSNOR expression, potentially via *S*-glutathionylation, as reported in *Neisseria meningitidis* EstD (Chen et al., 2013). These results raise the possibility that biofilm formation may be modulated by LMW thiols and nitric oxide through post-translational modifications such as *S*-glutathionylation or *S*-nitrosylation.

Transcriptomic analysis of the *gshA* mutant strain supports the central role of GSH in *A. baumannii* virulence. Upregulation of the phenylacetate (PAA) catabolic pathway aligns with reduced virulence observed in ATCC 17978 *gshA* mutant strains in the *Galleria mellonella* infection model. Similarly, *gshA* mutant strains in *P. aeruginosa* are less virulent in *Caenorhabditis elegans* and murine pneumonia models (Feinbaum et al., 2012; Michie et al., 2020), and *Salmonella enterica gshA* mutant strains show attenuated virulence in a murine model of acute infection (Song et al., 2013).

Overall, this work highlights the multifaceted roles of GSH in stress resistance, motility, biofilm formation, and virulence in an important ESKAPE pathogen. Further studies are needed to dissect the regulatory and signaling mechanisms by which LMW thiols, including GSH, modulate these critical cellular processes. Understanding these pathways may reveal new targets for therapeutic intervention against *A. baumannii*.

## CRediT authorship contribution statement

**Jason M. Thomas:** Conceptualization, Methodology, Software, Validation, Formal analysis, Investigation, Data curation, Writing – original draft, Writing – review & editing, Visualization. **Pegah Mosharaf Ghahfarokhy:** Methodology, Software, Validation, Formal analysis, Investigation, Data curation, Writing – original draft, Writing – review & editing, Visualization. **Samantha Gabrielle Perrotti Rivera:** Methodology, Investigation. **Clarissa J. Nobile:** Conceptualization, Methodology, Formal analysis, Resources, Data curation, Writing – original draft, Writing – review & editing, Visualization, Supervision, Project administration, Funding acquisition. **Mamta Rawat:** Conceptualization, Methodology, Formal analysis, Resources, Data curation, Writing – original draft, Writing – review & editing, Visualization, Supervision, Project administration, Funding acquisition.

## Declaration of competing interest

The authors declare the following financial interests/personal relationships which may be considered as potential competing interests:

Clarissa J. Nobile and Mamta Rawat report financial support was provided by the National Institutes of Health. Mamta Rawat reports financial support was provided by the National Science Foundation. Clarissa J. Nobile reports a relationship with BioSynesis, Inc. that includes: board membership and equity or stocks. Other authors declare that they have no known competing financial interests or personal relationships that could have appeared to influence the work reported in this paper.

## Appendix Supplementary materials

Image, application 1

Image, application 2

## References

[bib0001] Ahmad I., Nadeem A., Mushtaq F., Zlatkov N., Shahzad M., Zavialov A.V., Wai S.N., Uhlin B.E. (2023). Csu pili dependent biofilm formation and virulence of Acinetobacter baumannii. NPJ. Biofilms. Microbiomes..

[bib0002] Allocati N., Federici L., Masulli M., Di Ilio C. (2009). Glutathione transferases in bacteria. FEBS. J..

[bib0003] Alquethamy S.F., Adams F.G., Naidu V., Khorvash M., Pederick V.G., Zang M., Paton J.C., Paulsen I.T., Hassan K.A., Cain A.K. (2019). The role of zinc efflux during Acinetobacter baumannii infection. ACS. Infect. Dis..

[bib0004] Arenas F.A., Díaz W.A., Leal C.A., Pérez-Donoso J.M., Imlay J.A., Vásquez C.C. (2010). The *Escherichia coli* btuE gene, encodes a glutathione peroxidase that is induced under oxidative stress conditions. Biochem. Biophys. Res. Commun..

[bib0005] Baugh A.C., Momany C., Neidle E.L. (2023). Versatility and complexity: common and uncommon facets of LysR-type transcriptional regulators. Annu. Rev. Microbiol..

[bib0006] Bhuiyan M.S., Ellett F., Murray G.L., Kostoulias X., Cerqueira G.M., Schulze K.E., Mahamad Maifiah M.H., Li J., Creek D.J., Lieschke G.J (2016). Acinetobacter baumannii phenylacetic acid metabolism influences infection outcome through a direct effect on neutrophil chemotaxis. Proc. Natl Acad. Sci..

[bib0007] Blaschke U., Skiebe E., Wilharm G. (2021). Novel genes required for surface-associated motility in Acinetobacter baumannii. Curr. Microbiol..

[bib0008] Boll J.M., Tucker A.T., Klein D.R., Beltran A.M., Brodbelt J.S., Davies B.W., Trent M.S. (2015). Reinforcing lipid A acylation on the cell surface of Acinetobacter baumannii promotes cationic antimicrobial peptide resistance and desiccation survival. mBio.

[bib0009] Chandrangsu P., Loi V.V., Antelmann H., Helmann J.D. (2018). The role of bacillithiol in gram-positive firmicutes. Antioxid. Redox. Signal..

[bib0010] Chardonnet S., Sakr S., Cassier-Chauvat C., Le Maréchal P., Chauvat F., Lemaire S.D., Decottignies P. (2015). First proteomic study of S-glutathionylation in cyanobacteria. J. Proteome Res..

[bib0011] Chen N.H., Counago R.M., Djoko K.Y., Jennings M.P., Apicella M.A., Kobe B., McEwan A.G. (2013). A glutathione-dependent detoxification system is required for formaldehyde resistance and optimal survival of Neisseria meningitidis in biofilms. Antioxid. Redox. Signal..

[bib0012] Cuthbertson L., Nodwell J.R. (2013). The TetR family of regulators. Microbiol. Mol. Biol. Rev..

[bib0013] Echenique J.R., Dorsey C.W., Patrito L.C., Petroni A., Tolmasky M.E., Actis L.A. (2001). Acinetobacter baumannii has two genes encoding glutathione-dependent formaldehyde dehydrogenase: evidence for differential regulation in response to iron. Microbiology.

[bib0014] Fahey R., Brown W., Adams W., Worsham M. (1978). Occurrence of glutathione in bacteria. J. Bacteriol..

[bib0015] Fahey R.C., Newton G.L. (1987). Methods in Enzymology.

[bib0016] Fang Z., Dos Santos P.C (2015). Protective role of bacillithiol in superoxide stress and Fe–S metabolism in Bacillus subtilis. Microbiologyopen..

[bib0017] Feinbaum R.L., Urbach J.M., Liberati N.T., Djonovic S., Adonizio A., Carvunis A.-R., Ausubel F.M. (2012). Genome-wide identification of Pseudomonas aeruginosa virulence-related genes using a Caenorhabditis elegans infection model. PLoS. Pathog..

[bib0018] Ferguson G.P., Munro A.W., Douglas R.M., McLaggan D., Booth I.R. (1993). Activation of potassium channels during metabolite detoxification in *Escherichia coli*. Mol. Microbiol..

[bib0019] Gallagher L.A., Ramage E., Weiss E.J., Radey M., Hayden H.S., Held K.G., Huse H.K., Zurawski D.V., Brittnacher M.J., Manoil C. (2015). Resources for genetic and genomic analysis of emerging pathogen Acinetobacter baumannii. J. Bacteriol..

[bib0020] Gebhardt M.J., Gallagher L.A., Jacobson R.K., Usacheva E.A., Peterson L.R., Zurawski D.V., Shuman H.A. (2015). Joint transcriptional control of virulence and resistance to antibiotic and environmental stress in Acinetobacter baumannii. mBio.

[bib0021] Gomez M.J., Neyfakh A.A. (2006). Genes involved in intrinsic antibiotic resistance of Acinetobacter baylyi. Antimicrob. Agents Chemother.

[bib0022] Gulati M., Thomas J.M., Ennis C.L., Hernday A.D., Rawat M., Nobile C.J. (2024). The bacillithiol pathway is required for biofilm formation in Staphylococcus aureus. Microb. Pathog..

[bib0023] Haft D.H., Loftus B.J., Richardson D.L., Yang F., Eisen J.A., Paulsen I.T., White O. (2001). TIGRFAMs: a protein family resource for the functional identification of proteins. Nucleic. Acids. Res..

[bib0024] Harding C.M., Hennon S.W., Feldman M.F. (2018). Uncovering the mechanisms of Acinetobacter baumannii virulence. Nat. Rev. Microbiol..

[bib0025] Hartl J., Kiefer P., Kaczmarczyk A., Mittelviefhaus M., Meyer F., Vonderach T., Hattendorf B., Jenal U., Vorholt J.A. (2020). Untargeted metabolomics links glutathione to bacterial cell cycle progression. Nat. Metab..

[bib0026] Heindorf M., Kadari M., Heider C., Skiebe E., Wilharm G. (2014). Impact of Acinetobacter baumannii superoxide dismutase on motility, virulence, oxidative stress resistance and susceptibility to antibiotics. PLoS. One.

[bib0027] Helbig K., Bleuel C., Krauss G.J., Nies D.H. (2008). Glutathione and transition-metal homeostasis in *Escherichia coli*. J. Bacteriol..

[bib72] Helmann J.D. (2011). Bacillithiol, a new player in bacterial redox homeostasis. ARS..

[bib0028] Hider R.C., Kong X.L. (2011). Glutathione: a key component of the cytoplasmic labile iron pool. Biometals.

[bib0029] Hooppaw A.J., McGuffey J.C., Di Venanzio G., Ortiz-Marquez J.C., Weber B.S., Lightly T.J., van Opijnen T., Scott N.E., Cardona S.T., Feldman M.F. (2022). The phenylacetic acid catabolic pathway regulates antibiotic and oxidative stress responses in Acinetobacter. mBio.

[bib0030] Jacobs A.C., Thompson M.G., Gebhardt M., Corey B.W., Yildirim S., Shuman H.A., Zurawski D.V. (2014). Genetic manipulation of Acinetobacter baumannii. Curr. Protoc. Microbiol..

[bib0031] Johnson T., Newton G.L., Fahey R.C., Rawat M. (2009). Unusual production of glutathione in actinobacteria. Arch. Microbiol..

[bib0032] Karp P.D., Billington R., Caspi R., Fulcher C.A., Latendresse M., Kothari A., Keseler I.M., Krummenacker M., Midford P.E., Ong Q. (2019). The BioCyc collection of microbial genomes and metabolic pathways. Brief. Bioinform..

[bib0033] Knoke L.R., Muskietorz M., Kühn L., Leichert L.I. (2025). The ABC transporter Opp imports reduced glutathione, while Gsi imports glutathione disulfide in *Escherichia coli*. Redox. Biol..

[bib0034] König P., Averhoff B., Müller V. (2020). A first response to osmostress in Acinetobacter baumannii: transient accumulation of K+ and its replacement by compatible solutes. Environ. Microbiol. Rep..

[bib0036] Liao Y., Smyth G.K., Shi W. (2019). The R package Rsubread is easier, faster, cheaper and better for alignment and quantification of RNA sequencing reads. Nucleic. Acids. Res..

[bib0037] Liu L., Hausladen A., Zeng M., Que L., Heitman J., Stamler J.S. (2001). A metabolic enzyme for S-nitrosothiol conserved from bacteria to humans. Nature.

[bib0038] Lostroh C.P., Voyles B.A. (2010). Acinetobacter baylyi starvation-induced genes identified through incubation in long-term stationary phase. Appl. Environ. Microbiol..

[bib0039] Marcén M., Cebrián G., Ruiz-Artiga V., Condón S., Mañas P. (2019). Protective effect of glutathione on *Escherichia coli* cells upon lethal heat stress. Food Res. Int..

[bib0040] Masip L., Veeravalli K., Georgiou G. (2006). The many faces of glutathione in bacteria. Antioxid. Redox. Signal..

[bib0041] Meister A. (1995). Methods in Enzymology.

[bib0042] Michie K.L., Dees J.L., Fleming D., Moustafa D.A., Goldberg J.B., Rumbaugh K.P., Whiteley M. (2020). Role of Pseudomonas aeruginosa glutathione biosynthesis in lung and soft tissue infection. Infect. Immun..

[bib0043] Mühlenhoff U., Braymer J.J., Christ S., Rietzschel N., Uzarska M.A., Weiler B.D., Lill R. (2020). Glutaredoxins and iron-sulfur protein biogenesis at the interface of redox biology and iron metabolism. Biol. Chem..

[bib0044] Newton G., Fahey R., Cohen G., Aharonowitz Y. (1993). Low-molecular-weight thiols in streptomycetes and their potential role as antioxidants. J. Bacteriol..

[bib0045] Newton G.L., Buchmeier N., Fahey R.C. (2008). Biosynthesis and functions of mycothiol, the unique protective thiol of actinobacteria. Microbiol. Mol. Biol. Rev..

[bib0046] Newton G.L., Fahey R.C. (1987). Methods in Enzymology.

[bib0047] Pakharukova N., Tuittila M., Paavilainen S., Malmi H., Parilova O., Teneberg S., Knight S.D., Zavialov A.V. (2018). Structural basis for Acinetobacter baumannii biofilm formation. Proc. Natl Acad. Sci..

[bib0048] Park S., Imlay J.A. (2003). High levels of intracellular cysteine promote oxidative DNA damage by driving the Fenton reaction. J. Bacteriol..

[bib0035] Pokhrel P., Dinh H., Li L., Hassan K.A., Cain A.K., Paulsen I.T. (2023). Identification of a novel LysR Family transcriptional regulator controlling acquisition of sulfur sources in Acinetobacter. Microb. Physiol..

[bib0049] Potter A.J., Trappetti C., Paton J.C. (2012). Streptococcus pneumoniae uses glutathione to defend against oxidative stress and metal ion toxicity. J. Bacteriol..

[bib0050] Rajkarnikar A., Strankman A., Duran S., Vargas D., Roberts A.A., Barretto K., Upton H., Hamilton C.J., Rawat M. (2013). Analysis of mutants disrupted in bacillithiol metabolism in Staphylococcus aureus. Biochem. Biophys. Res. Commun..

[bib0051] Rawat M., Johnson C., Cadiz V., Av-Gay Y. (2007). Comparative analysis of mutants in the mycothiol biosynthesis pathway in Mycobacterium smegmatis. Biochem. Biophys. Res. Commun..

[bib0052] Reyes A.M., Pedre B., De Armas M.I., Tossounian M.-A., Radi R., Messens J., Trujillo M. (2018). Chemistry and redox biology of mycothiol. Antioxid. Redox. Signal..

[bib0053] Riccillo P.M., Muglia C.I., De Bruijn F.J., Roe A.J., Booth I.R., Aguilar O.M. (2000). Glutathione is involved in environmental stress responses in rhizobium tropici, including acid tolerance. J. Bacteriol..

[bib0054] Robinson M.D., McCarthy D.J., Smyth G.K. (2010). edgeR: a bioconductor package for differential expression analysis of digital gene expression data. Bioinformatics..

[bib0055] Rumbo-Feal S., Gomez M.J., Gayoso C., Álvarez-Fraga L., Cabral M.P., Aransay A.M., Rodriguez-Ezpeleta N., Fullaondo A., Valle J., Tomás M. (2013). Whole transcriptome analysis of Acinetobacter baumannii assessed by RNA-sequencing reveals different mRNA expression profiles in biofilm compared to planktonic cells. PLoS. One.

[bib0056] Sao Emani C., Williams M., Wiid I., Baker B. (2018). The functional interplay of low molecular weight thiols in Mycobacterium tuberculosis. J. Biomed. Sci..

[bib0057] Sarshar M., Behzadi P., Scribano D., Palamara A.T., Ambrosi C. (2021). Acinetobacter baumannii: an ancient commensal with weapons of a pathogen. Pathogens.

[bib0058] Schmittgen T.D., Livak K.J. (2008). Analyzing real-time PCR data by the comparative CT method. Nat. Protoc..

[bib0059] Song M., Husain M., Jones-Carson J., Liu L., Henard C.A., Vázquez-Torres A. (2013). Low-molecular-weight thiol-dependent antioxidant and antinitrosative defences in S almonella pathogenesis. Mol. Microbiol..

[bib0060] Sun D., Crowell S.A., Harding C.M., De Silva P.M., Harrison A., Fernando D.M., Mason K.M., Santana E., Loewen P.C., Kumar A. (2016). KatG and KatE confer Acinetobacter resistance to hydrogen peroxide but sensitize bacteria to killing by phagocytic respiratory burst. Life Sci..

[bib0061] Tomlinson B.R., Denham G.A., Torres N.J., Brzozowski R.S., Allen J.L., Jackson J.K., Eswara P.J., Shaw L.N. (2022). Assessing the role of cold-shock protein C: a novel regulator of Acinetobacter baumannii biofilm formation and virulence. Infect. Immun..

[bib0062] Upmanyu K., Haq Q.M.R., Singh R. (2022). Factors mediating Acinetobacter baumannii biofilm formation: opportunities for developing therapeutics. Curr. Res. Microb. Sci..

[bib0063] Van Laar T.A., Esani S., Birges T.J., Hazen B., Thomas J.M., Rawat M. (2018). Pseudomonas aeruginosa gshA mutant is defective in biofilm formation, swarming, and pyocyanin production. mSphere.

[bib0064] Vargas D., Hageman S., Gulati M., Nobile C.J., Rawat M. (2016). S-nitrosomycothiol reductase and mycothiol are required for survival under aldehyde stress and biofilm formation in mycobacterium smegmatis. IUBMB Life.

[bib0065] Vázquez-López R., Solano-Gálvez S.G., Juárez Vignon-Whaley J.J., Abello Vaamonde J.A., Padró Alonzo L.A., Rivera Reséndiz A., Muleiro Álvarez M., Vega López E.N., Franyuti-Kelly G., Álvarez-Hernández D.A (2020). Acinetobacter baumannii resistance: a real challenge for clinicians. Antibiotics.

[bib0066] Walsh B.J., Wang J., Edmonds K.A., Palmer L.D., Zhang Y., Trinidad J.C., Skaar E.P., Giedroc D.P. (2020). The response of Acinetobacter baumannii to hydrogen sulfide reveals two independent persulfide-sensing systems and a connection to biofilm regulation. mBio.

[bib0067] Whiteway C., Breine A., Philippe C., Van der Henst C. (2022). Acinetobacter baumannii. Trends. Microbiol..

[bib0068] Wongsaroj L., Saninjuk K., Romsang A., Duang-Nkern J., Trinachartvanit W., Vattanaviboon P., Mongkolsuk S. (2018). Pseudomonas aeruginosa glutathione biosynthesis genes play multiple roles in stress protection, bacterial virulence and biofilm formation. PLoS. One.

[bib0070] Zhang Y., Zhang C., Du X., Zhou Y., Kong W., Lau G.W., Chen G., Kohli G.S., Yang L., Wang T. (2019). Glutathione activates type III secretion system through Vfr in Pseudomonas aeruginosa. Front. Cell Infect. Microbiol..

