# Supplementary material for: The glutathione pathway is required for biofilm formation in Acinetobacter baumannii
Source: Curr Res Microb Sci. 2026 Jan 29;10:100562. doi: 10.1016/j.crmicr.2026.100562 (PMC12914303; doi:10.1016/j.crmicr.2026.100562)
Supplement: Supplementary file 1 [file mmc1.pdf]

Fig. S1

```
ABUW_2594     ------------MRALTYHGAEDVRVETVADPIIQEPDDIILRVTATAICGSDLHLYRGK
BTH_I0686     ---------MKTKAAIAWKAGEPLTIEEV-DLEGPRAGEVLIEVKATGICHTDYYTLSGA
PA3629        --------MIKSRAAVAFAPNKPLEIVEV-DVAPPQKGEVLVRIVATGVCHTDAYTLSGQ
ABUW_2057     ---------MKSRAAVAFAPGKPLEIVEV-DVAPPKAGEVLIKITHTGVCHTDAFTLSGD
NMB1304       MEMKQADSTIKSRAAVAFAPNQPLQIVEI-DVEMPRKGEVLIRNTHTGVCHTDAFTLSGS
                            *:::   : : :  : *    . .::::.   *.:* :* .   *

ABUW_2594     IPKTEHGDIFGHEFMGIVEEVGPAVTAVKKGDRVIVPFVIACGECFFCQLDLTAACETTN
BTH_I0686     DPEGLFPAILGHEGAGVVVDVGPGVTLKKGDHVIPLYTPECRECKFCLSRKTNLCQKIR
PA3629        DSEGVFPCILGHEGGGIVEAVGEGVTSLQVGDHVIPLYTAECGKCKFCLSGKTNLCQAVR
ABUW_2057     DPEGVFPAILGHEGAGVVVEVGEGVTSVQPGDHVIPLYTAECKECLFCKSGKTNLCVAVR
NMB1304       DPEGVFPVVLGHEGAGVVVAVGEGVSSVKPGDHVIPLYTAECGECEFCCSGKTNLCVSVR
                :  .  ::***  *:*  ** .* ::: **:**  :.  * :* **    *  *   .

ABUW_2594     TGRGAIINKKQIPPGAALFGYSHL----YGGVPGGQAEYVRVPKANKGPFKVPGSLSDEK
BTH_I0686     ATQGR----GLMPDATSRFSIGGKPIFHYMGT-STFSNYIVVPEIAV--AKVREDAPFDK
PA3629        ATQGK----GLMPDGTSRFSYKGEPVFHYMGC-STFSEYTVLPEISL--AKIPKDAPLEK
ABUW_2057     ATQGK----GVMPDGTTRFSYNGQPIYHYMGC-STFSEYTVVAEVSL--AKINPEANHEQ
NMB1304       DTQGK----GLMPDGTTRFSYQGQPIYHYMGC-STFSEYSVVAEVSL--AKINPEANHEQ
                :*       :* .:: *.        * *   .  ::*  : :      *:  .   ::

ABUW_2594     VLFLTDILPTA-WQAVNNAQVKRGSTVAIYGAGPVGLLSAACAKMLGVEKIFMVDHNEYR
BTH_I0686     ICYIGCGVTTGVGAVVYSAKVEAGANVVVFGLGGIGLNVIQGARMVGADKIIGVDINPKR
PA3629        VCLLGCGVTTGIGAVLNTAKVEEGATVAIFGLGGIGLAAIIGAKMAKAARIIAVDINPGK
ABUW_2057     VCLLGCGVTTGIGAVHNTAKVQEGDSVAVFGLGGIGLAVVQGARQAKAGRIIVVDTNPDK
NMB1304       VCLLGCGVTTGIGAVHNTAKVQEGDSVAVFGLGAIGLAVVQGARQAKAGRIIAIDTNPSK
              :  :   : *.   .  .*:*: * .*.::* * :**     *:   . :*: :* *  :

ABUW_2594     LNYAAATYDAIPVNFDEVDAA--EFIIQNTDNYRGVDAVIDAIGFEAKGSVIETVLTNLK
BTH_I0686     VELARKFGMTHFVNPNEVENV-VDHIVQLT--DGGADYSFECI-----GNVK--------
PA3629        FDIARELGATDFINPKDYDKPIQDVIVELT--DGGVDYSFECV-----GNVQ--------
ABUW_2057     FELAKQFGATDFLNPKDYDQPIQQVIVEMT--GWGVDHSFECI-----GNVN--------
NMB1304       FELAKQFGATDCLNPNDYDKPIKDVLLDIN--KWGIDHTFECI-----GNVN--------
              .: *     :  :* .: :    : ::: .    * *  ::.:     *.*

ABUW_2594     LEGSSGAALRQCIAAVRRGGV----VSVPG----VYAGPIHGFLFGDAFDKGLTFKMGQT
BTH_I0686     -------VMRQALECTHKGWGQSFIIGVAAAGEEISTRPF-QLVTGREW-KG--SAFGGA
PA3629        -------LMRAALECCHKGWGESVIIGVAGAGQEISTRPF-QLVTGRVW-RG--SAFGGV
ABUW_2057     -------VMRSALECAHRGWGQSVIIGVAGAGQEISTRPF-QLVTGRKW-LG--TAFGGV
NMB1304       -------VMRQALESAHRGWGQSIIIGVAGAGQEISTRPF-QLVTGRVW-KG--SAFGGV
                      :* .: . ::*      :.* .    : : *:  :: *  :  *    :* .

ABUW_2594     HVHQYLPQLLELIERGELTPETIITHRMKLEDAAEGYRIFNEREEDCRKVILLP   387
BTH_I0686     RGRTDVPKIVDWYMEGKINIDDLITHTLPLEKINDGFDLMKKGES-IRSVVLY-   368
PA3629        RGRSELPSYVEKAQKGEIPLDTFITHTMGLEDINEAFELMHEGKS-IRTVIHY-   370
ABUW_2057     KGRSQLPKMVEDAMKGDIQLEPFVTHTMPLQDINTAFDLMQEGKS-IRTVIHF-   369
NMB1304       KGRSELPKMVEDSMKGDIELEPFVTHTMTLDQINKAFDLMHEGKS-IRAVIHY-   378
              : :  :*. ::   .*.:  : ::** : *:.   .: :::: :.  * *:
```

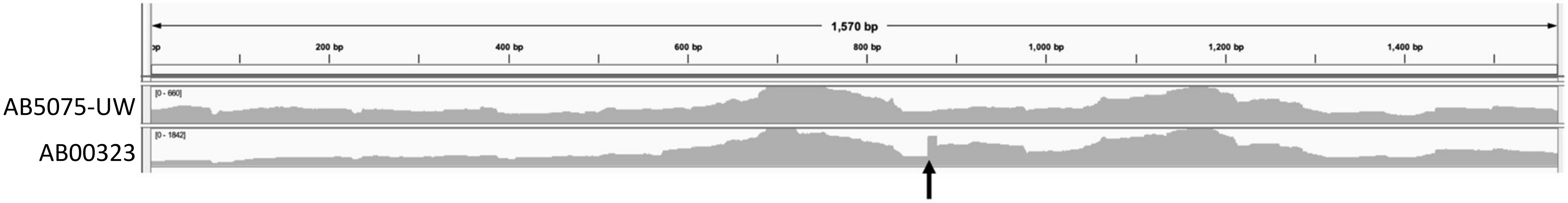
1,570 bp
200 bp
400 bp
600 bp
800 bp
1,000 bp
1,200 bp
1,400 bp
AB5075-UW
[0 - 660]
AB00323
[0 - 1842]

Fig. S2

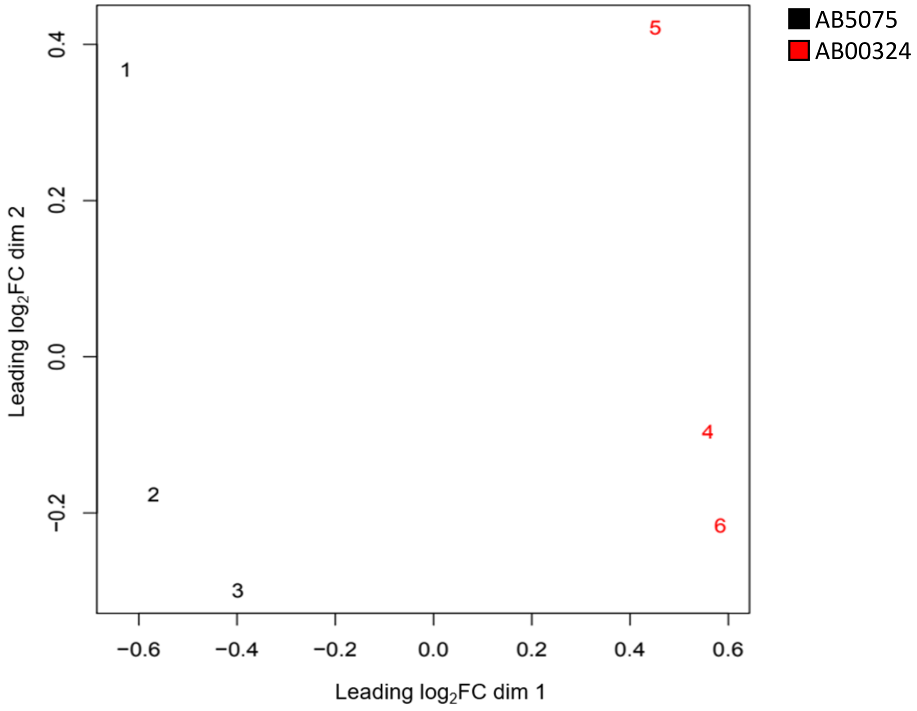

AB5075
AB00324
1
2
3
4
5
6
0.4
0.2
0.0
−0.2
Leading log2FC dim 2
−0.6
−0.4
−0.2
0.0
0.2
0.4
0.6
Leading log2FC dim 1

Fig. S3

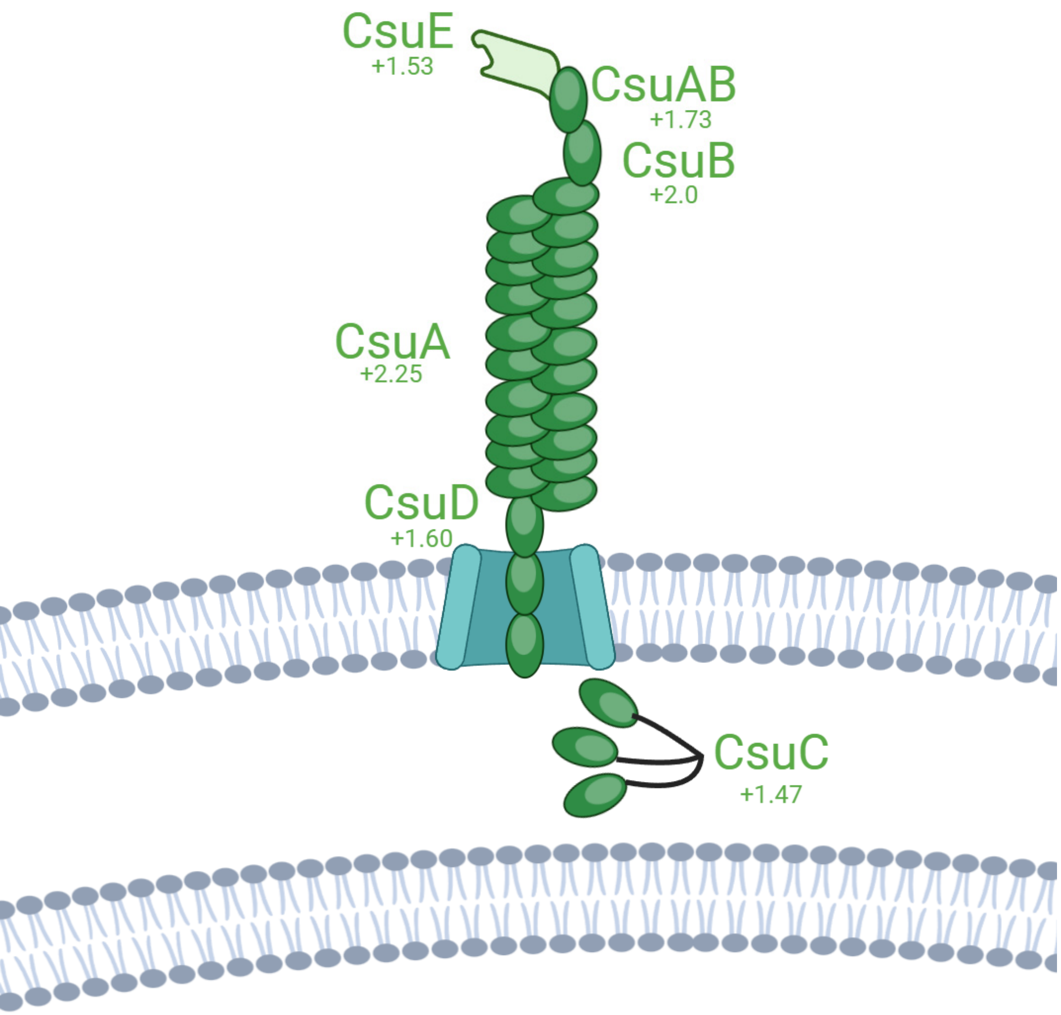

Fig. S4

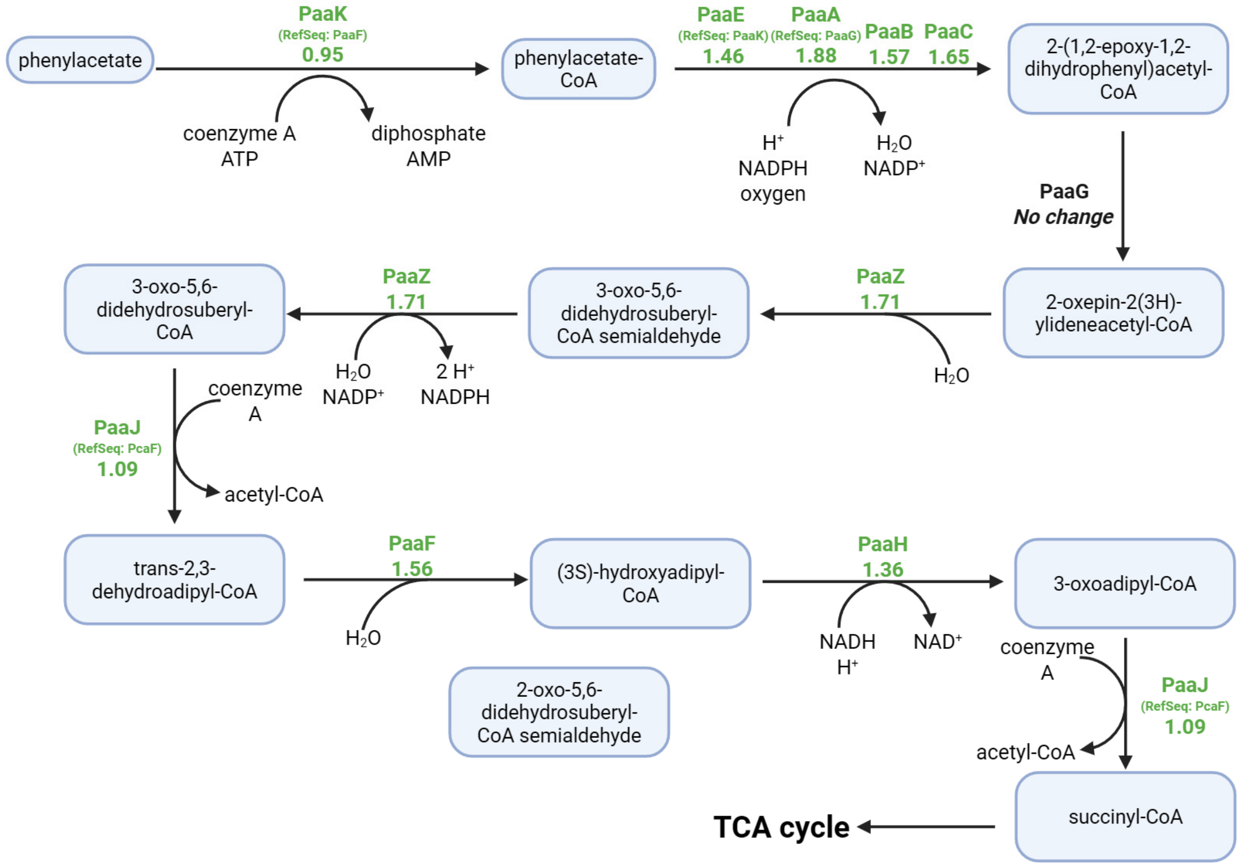

phenylacetate
PaaK
(RefSeq: PaaF)
0.95
coenzyme A
ATP
diphosphate
AMP
phenylacetate-CoA
PaaE
(RefSeq: PaaK)
1.46
PaaA
(RefSeq: PaaG)
1.88
PaaB
1.57
PaaC
1.65
H+
NADPH
oxygen
H2O
NADP+
2-(1,2-epoxy-1,2-dihydrophenyl)acetyl-CoA
PaaG
No change
2-oxepin-2(3H)-ylideneacetyl-CoA
PaaZ
1.71
H2O
3-oxo-5,6-didehydrosuberyl-CoA semialdehyde
PaaZ
1.71
H2O
NADP+
2 H+
NADPH
3-oxo-5,6-didehydrosuberyl-CoA
coenzyme A
PaaJ
(RefSeq: PcaF)
1.09
acetyl-CoA
trans-2,3-dehydroadipyl-CoA
PaaF
1.56
H2O
(3S)-hydroxyadipyl-CoA
PaaH
1.36
NADH
H+
NAD+
3-oxoadipyl-CoA
2-oxo-5,6-didehydrosuberyl-CoA semialdehyde
coenzyme A
PaaJ
(RefSeq: PcaF)
1.09
acetyl-CoA
succinyl-CoA
TCA cycle

Fig. S5

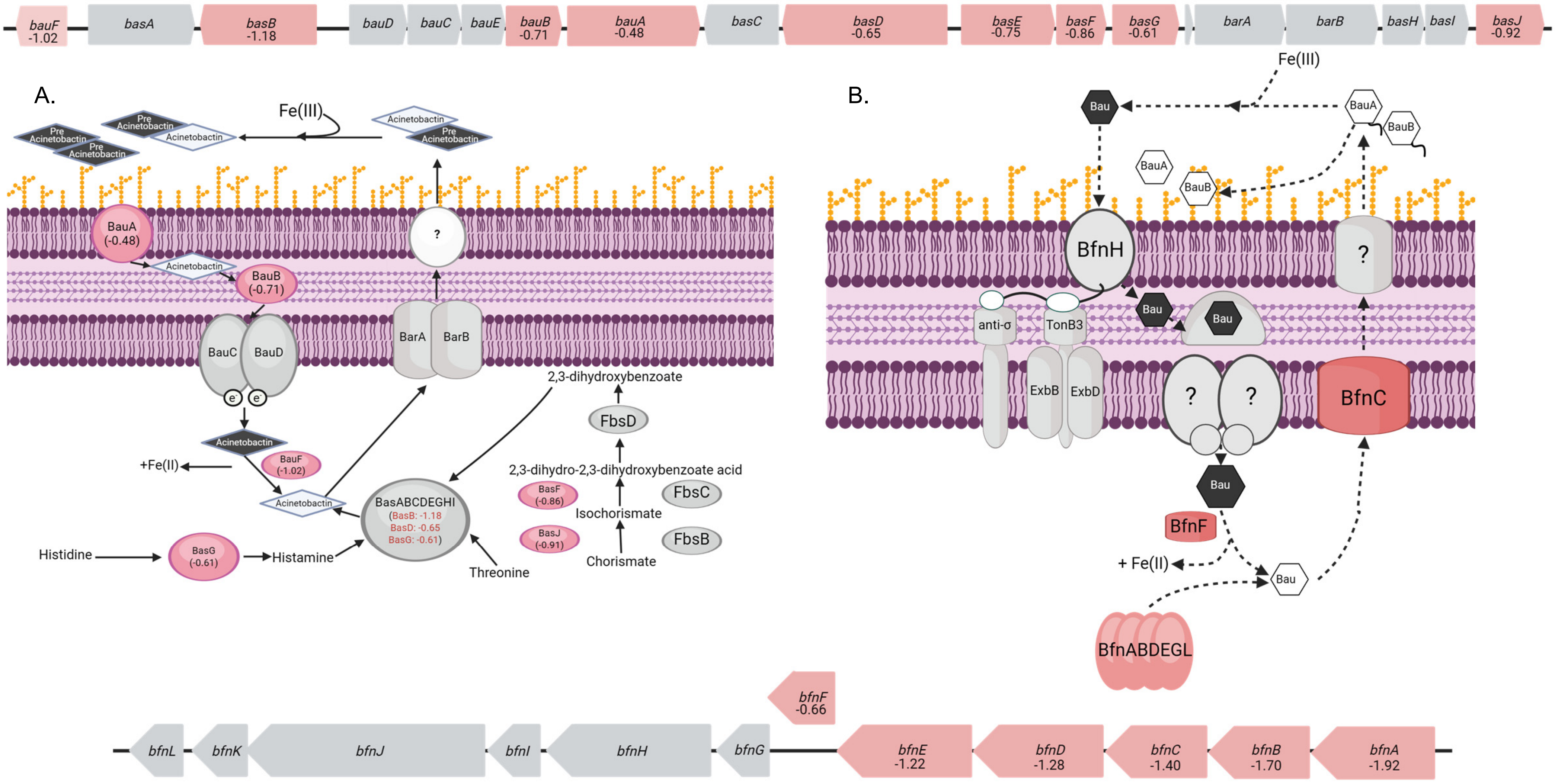

bauF -1.02
basA
basB -1.18
bauD
bauC
bauE
bauB -0.71
bauA -0.48
basC
basD -0.65
basE -0.75
basF -0.86
basG -0.61
barA
barB
basH
basI
basJ -0.92
A.
Fe(III)
Pre Acinetobactin
Acinetobactin
BauA (-0.48)
BauB (-0.71)
?
BarA
BarB
BauC
BauD
+Fe(II)
BauF (-1.02)
BasABCDEGHI
(BasB: -1.18
BasD: -0.65
BasG: -0.61)
Histidine
BasG (-0.61)
Histamine
Threonine
2,3-dihydroxybenzoate
FbsD
2,3-dihydro-2,3-dihydroxybenzoate acid
BasF (-0.86)
FbsC
Isochorismate
BasJ (-0.91)
FbsB
Chorismate
B.
Fe(III)
Bau
BauA
BauB
BfnH
anti-σ
TonB3
ExbB
ExbD
BfnC
BfnF
+ Fe(II)
BfnABDEGL
bfnF -0.66
bfnL
bfnK
bfnJ
bfnI
bfnH
bfnG
bfnE -1.22
bfnD -1.28
bfnC -1.40
bfnB -1.70
bfnA -1.92

Fig. S6

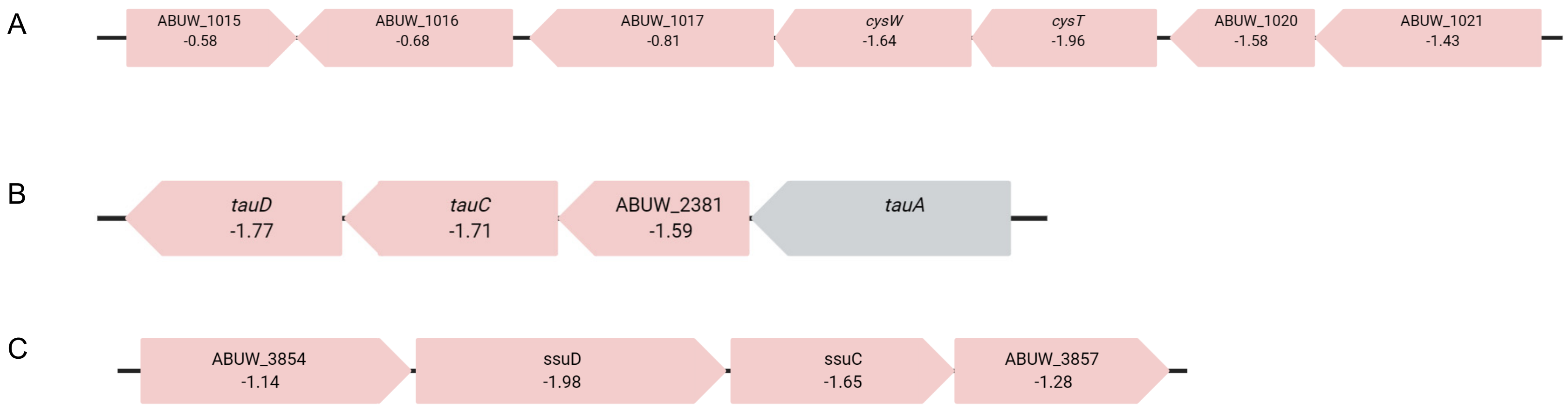
A
ABUW_1015
-0.58
ABUW_1016
-0.68
ABUW_1017
-0.81
cysW
-1.64
cysT
-1.96
ABUW_1020
-1.58
ABUW_1021
-1.43
B
tauD
-1.77
tauC
-1.71
ABUW_2381
-1.59
tauA
C
ABUW_3854
-1.14
ssuD
-1.98
ssuC
-1.65
ABUW_3857
-1.28

Fig.S7
