# Supplementary material for: The glutathione pathway is required for biofilm formation in Acinetobacter baumannii
Source: Curr Res Microb Sci. 2026 Jan 29;10:100562. doi: 10.1016/j.crmicr.2026.100562 (PMC12914303; doi:10.1016/j.crmicr.2026.100562)
Supplement: Supplementary file 2 [file mmc2.docx]

**Supplemental Materials**

**Supplemental Figure Legends**

**Fig. S1: Clustal Omega alignment of ABUW_2057 and ABUW_2594.** ABUW_2057 and ABUW_2594 amino acid sequences were aligned with other genes annotated as GSNORs with other putative GSNORs from *Burkholderia thailandensis* (BTH_I0686), *Pseudomonas aeruginosa* (PA3629) and a confirmed GSNOR from *Neisseria meningitidis* (NMB1304)(42).

**Fig. S2: Integrative Genome Viewer (IGV) visualization of coverage in *A. baumanii* AB5075-UW wildtype strain and AB00324 (*gshA^-^*) mutant strain**. The arrow denotes the site of transposon insertion in *gshA*.

**Fig. S3:** **Principal component analysis (PCA) plots of RNA-Seq samples**. *A. baumanii* AB5075-UW (wildtype); black numbers and AB00324 (*gshA^-^*); red numbers.

**Fig. S4**: **Csu fimbriae genes are upregulated in the *gshA* mutant strain.** Figure was adapted from Harding et al. (2017) (4).

**Fig. S5**: **Phenylacetate degradation pathway is upregulated in the *gshA* mutant strain**. Both BioCyc and RefSeq annotations are provided.

**Fig. S6**: **Iron acquisition in *A. baumannii***. A. Acinetobactin; B. Baumannoferrin. Downregulated genes in the *gshA* mutant strain are in red. Figure was adapted from Sheldon & Skaar (2020) (66).

**Fig.S7: Genes involved in sulfur assimilation are downregulated in *A. baumanni gshA* mutant strain**. A. Sulfate uptake; B. Taurine; C. Sulfonate uptake.

**Supplemental Tables**

**Table S1: Strains, plasmids, and primers used in the study**

| **Strain Name** | **Description** | **Reference** |
| --- | --- | --- |
| AB5075-UW | Wildtype strain | Gallagher *et al*., 2015 |
| AB5075-JT1 | Wildtype strain with pMJG120; Apra^R^ |  |
| AB00324 | AB5057-UW harboring a transposon insertion in ABUW_0114 (*gshA*); Tet^R^ |  |
| AB00412 | AB5057-UW harboring a transposon insertion in ABUW_0145 (*gshB*); Tet^R^ |  |
| AB05347 | AB5057-UW harboring a transposon insertion in ABUW_2057(*gsnoR1*); Tet^R^ |  |
| AB06842 | AB5057-UW harboring a transposon insertion in ABUW_2594 (*gsnoR2*); Tet^R^ |  |
| AB00324-JTgshA | AB00324 complemented with pMJG120.gshA; Tet^R^, Apra^R^ | This study |
| AB00412-JTgshB | AB00412 complemented with pMJG120.gshB; Tet^R^, Apra^R^ |  |
| AB05347-PMgsnoR1 | complemented with pMJG120.gsnoR1; Tet^R^, Apra^R^ |  |
| AB06842-PMgsnoR2 | complemented with pMJG120.gsnoR2; Tet^R^, Apra^R^ |  |
| **Plasmids** | **Description** |  |
| pMJG120 | Complementation plasmid; IPTG inducible pTac promoter; Apra^R^ | Gebhardt *et al*., 2015 |
| JtgshA | pMJG120 harboring the *gshA* wildtype sequence; Apra^R^ | This study |
| JTgshB | pMJG120 harboring the *gshB* wildtype sequence; Apra^R^ |  |
| PMgsnoR1 | pMJG120 harboring the *gsnoR1* wildtype sequence; Apra^R^ |  |
| PMgsnoR1 | pMJG120 harboring the *gsnoR2* wildtype sequence; Apra^R^ |  |
| **Primers for Strain Construction** | **Sequence (5’-3’)** | **Reference** |
| Pgro-172 (Transposon specific primer) | TGAGCTTTTTAGCTCGACTAATCCAT | Gallagher *et al*., 2015 |
| *gshA* Fwd Confirmation Primer | GTCAACCCACTACACCAT | This study |
| *gshA* Rev Confirmation Primer | TTGTTCTTGCTGCTGTAAAG |  |
| *gshB* Fwd Confirmation Primer | ATCCGATTGAAACCGTAAATCTG |  |
| *gshB* Rev Confirmation Primer | ATGCTGGACGACCAGCTTCT |  |
| *gshA* Fwd Complementation primer | GATCCAGAATTCATGAGTCAACCCACTACA  (*EcoR*I restriction site) |  |
| *gshA*^-^Rev Complementation primer | GTCAAGCTTTTATCTATATTGTTCAAG  (*Hind*III restriction site) |  |
| *gshB*^-^Fwd Complementation primer | GATCCAGAATTCATGCGTGTACTTGTCGTCA  (*EcoR*I restriction site) |  |
| *gshB*^-^Rev Complementation primer | GTCAAGCTTTTATGCTGGACGACCAGC  (*Hind*III restriction site) |  |
| *gsnoR1* Fwd Complementation primer | GATCCAGAATTCATGAAATCTCGTGCAGCTGTCG |  |
| *gsnoR1*^-^Rev Complementation primer | GTCAAGCTTTTAGAAATGAATAACAGTACGAAT |  |
| *gsnoR2*^-^ Fwd Complementation primer | GATCGAATTCATGCGCGCTCTTACTTACCACGGT |  |
| *gsnoR2* ^-^Rev Complementation primer | GTCAAGCTTTAAGGAAGCAGAATCACCTTACGGC |  |
| **Primers for qPCR** | **Sequence (5’-3’)** |  |
| RT_AB_16s_F  (Housekeeping gene) | CTTCGGACCTTGCGCTAATA | Sun *et al*., 2016 |
| RT_AB_16s_R | ATCCTCTCAGACCCGCTACA |  |
| RT_AB_gyrB_F  *(Housekeeping gene)* | TCTCTAGTCAGGAAGTGGGTACATT | Funston *et al*., 2016 |
| RT_AB_gyrB_R | GGTTATATTCTTCACGGCCAAT |  |
| RT_AB_gshA_Fwd | AGCTCTGGCCGTTGTCTATG | This study |
| RT_AB_gshA_Rev | ACGGCGACCATAACGAATAC |  |
| RT_AB_gshB_Fwd | TCCTGAATTACAGGTGCCAAC |  |
| RT_AB_gshB_Rev | GATCACATCACCGTGCTCAG |  |
| RT_AB_2316_Fwd | ATGATGTTCGGTGCTTTGAC |  |
| RT_AB_2316_Rev | CCCATAGCCAAGTGTGCAG |  |
| RT_AB_2096_Fwd | CCCACAAGGCACCTTAGC |  |
| RT_AB_2096_Rev | TCCTTACCTTCCGCAATGAG |  |
| RT_AB_1572_Fwd | ATTACAGGTGGTGGCTCTGG |  |
| RT_AB_1572_Rev | TGCTGCTCACCTGCTTCTAC |  |
| RT_AB_1477_Fwd | CTGAGATTCAGCAGCGTGAC |  |
| RT_AB_1477_Rev | CAAGCGTGGTAATTGCATTG |  |
| RT_AB_1481_Fwd | ACCTGACGAAGAGTGGATGC |  |
| RT_AB_1481_Rev | CGATGGTGCATAATGTCGAG |  |
| RT_AB_2535_Fwd | AGATGGCACAAGATGCAGTG |  |
| RT_AB_2535_Rev | TGGAGAATGTTCATCGCTTG |  |

**Table S2: Transcriptome assembly statistics of *A. baumanii* AB5075-UW wildtype strain and AB00324 *gshA* mutant strain samples**

| **Sample** | **Total Reads** | **Mapped Reads** | **Mapping Percentage** | **Reference Genome** |
| --- | --- | --- | --- | --- |
| AB5075-UW 1 | 9,613,413 | 8,622,208 | 89.7% | ABUW RefSeq |
| AB5075-UW 2 | 9,723,748 | 8,676,528 | 89.2% | GCF_000963815.1 |
| AB5075-UW 3 | 9,298,911 | 8,343,388 | 89.7% |  |
| AB00324 1 | 9,552,621 | 8,657,232 | 90.6% |  |
| AB00324 2 | 8,988,985 | 8,202,842 | 91.3% |  |
| AB00324 3 | 8,629,789 | 7,861,639 | 91.1% |  |

**Table S3: Upregulated genes with log_2_FC>= 1.0 identified in the AB00324 *gshA* mutant strain compared to the *A. baumanii* AB5075-UW wildtype strain.**

| **Gene** | **2nd Accession** | **Product** | **log_2_FC*** | **Adj P Val** |
| --- | --- | --- | --- | --- |
| ABUW_RS07200 | ABUW_1477 | sodium/glutamate symporter | 3.2 | 1.22E-07 |
| ABUW_RS07220 | ABUW_1481 | SDR family oxidoreductase | 2.4 | 3.22E-05 |
| ABUW_RS07225 | ABUW_1482 | SRPBCC family protein | 2.3 | 4.20E-05 |
| ABUW_RS07255 | ABUW_1488 | fimbrial major subunit CsuA/B family protein | 2.3 | 2.24E-03 |
| ABUW_RS07280 | ABUW_1493 | hypothetical protein | 2.1 | 3.83E-02 |
| ABUW_RS07205 | ABUW_1478 | cyclase family protein | 2.1 | 3.97E-05 |
| ABUW_RS07260 | ABUW_1489 | Csu fimbrial biogenesis protein CsuB | 2.0 | 3.77E-05 |
| ABUW_RS07215 | ABUW_1480 | aldo/keto reductase | 2.0 | 3.22E-05 |
| ABUW_RS12325 | ABUW_2535 | 1,2-phenylacetyl-CoA epoxidase subunit A | 1.9 | 3.97E-05 |
| ABUW_RS02185 | ABUW_0445 | tRNA-Ser | 1.8 | 5.32E-03 |
| ABUW_RS07195 | ABUW_1476 | alpha/beta fold hydrolase | 1.8 | 3.22E-05 |
| ABUW_RS05445 | ABUW_1113 | alpha-keto acid decarboxylase family protein | 1.8 | 1.98E-04 |
| ABUW_RS07720 | ABUW_1581 | potassium-transporting ATPase subunit KdpA | 1.7 | 2.43E-04 |
| ABUW_RS07250 | ABUW_1487 | Csu fimbrial major subunit CsuAB | 1.7 | 2.07E-04 |
| ABUW_RS12330 | ABUW_2536 | phenylacetic acid degradation bifunctional protein PaaZ | 1.7 | 8.76E-05 |
| ABUW_RS12315 | ABUW_2533 | phenylacetate-CoA oxygenase subunit PaaC | 1.7 | 4.23E-04 |
| ABUW_RS12310 | ABUW_2532 | phenylacetate-CoA oxygenase subunit PaaJ | 1.6 | 1.33E-04 |
| ABUW_RS07230 | ABUW_1483 | ATP-binding protein | 1.6 | 1.20E-04 |
| ABUW_RS07270 | ABUW_1491 | Csu fimbrial biogenesis usher protein CsuD | 1.6 | 8.25E-05 |
| ABUW_RS12320 | ABUW_2534 | 1,2-phenylacetyl-CoA epoxidase subunit B | 1.6 | 3.18E-04 |
| ABUW_RS12300 | ABUW_2530 | enoyl-CoA hydratase | 1.6 | 1.44E-03 |
| ABUW_RS07275 | ABUW_1492 | Csu fimbrial tip adhesin CsuE | 1.5 | 2.92E-04 |
| ABUW_RS13400 | ABUW_2760 | pirin family protein | 1.5 | 6.72E-05 |
| ABUW_RS07210 | ABUW_1479 | LysR family transcriptional regulator | 1.5 | 4.20E-05 |
| ABUW_RS07265 | ABUW_1490 | Csu fimbrial biogenesis chaperone CsuC | 1.5 | 8.99E-05 |
| ABUW_RS12305 | ABUW_2531 | phenylacetate-CoA oxygenase/reductase subunit PaaK | 1.5 | 6.71E-04 |
| ABUW_RS07235 | ABUW_1484 | FadR family transcriptional regulator | 1.4 | 1.73E-03 |
| ABUW_RS07725 | ABUW_1582 | potassium-transporting ATPase subunit KdpB | 1.4 | 3.36E-04 |
| ABUW_RS12295 | ABUW_2529 | 2-(1,2-epoxy-1,2-dihydrophenyl)acetyl-CoA isomerase | 1.4 | 2.09E-03 |
| ABUW_RS05450 | ABUW_1114 | amino acid permease | 1.4 | 1.79E-04 |
| ABUW_RS12290 | ABUW_2528 | 3-hydroxyacyl-CoA dehydrogenase | 1.4 | 3.86E-04 |
| ABUW_RS10215 | ABUW_2103 | hypothetical protein | 1.3 | 1.81E-04 |
| ABUW_RS08725 | ABUW_1792 | cytochrome bd biosynthesis protein | 1.2 | 4.01E-04 |
| ABUW_RS12285 | ABUW_2527 | 3-oxoadipyl-CoA thiolase | 1.1 | 9.25E-04 |
| ABUW_RS07240 | ABUW_1485 | hypothetical protein | 1.1 | 4.11E-04 |
| ABUW_RS15025 | ABUW_3095 | tRNA-Gln | 1.1 | 1.78E-03 |
| ABUW_RS08745 | ABUW_1796 | hypothetical protein | 1.0 | 5.10E-04 |
| ABUW_RS09405 | ABUW_1931 | MFS transporter | 1.0 | 3.79E-03 |
| ABUW_RS19465 | ABUW_4097 | hypothetical protein | 1.0 | 4.90E-03 |

**Table S4: Downregulated genes with log_2_FC<= -1.0 identified in the AB00324 *gshA* mutant strain compared to the *A. baumanii* AB5075-UW wildtype strain.**

| **Gene** | **2nd Accession** | **Product** | **log_2_FC*** | **Adj P Val** |
| --- | --- | --- | --- | --- |
| ABUW_RS10185 | ABUW_2097 | CoA transferase subunit B, ScoB , AtoB | -3.3 | 3.22E-05 |
| ABUW_RS10180 | ABUW_2096 | CoA transferase subunit A ScoA, AtoD | -3.2 | 8.80E-07 |
| ABUW_RS11265 | ABUW_2316 | EamA family transporter | -2.6 | 3.77E-05 |
| ABUW_RS07680 | ABUW_1572 | 3-hydroxyacyl-CoA dehydrogenase | -2.6 | 4.22E-05 |
| ABUW_RS07685 | ABUW_1573 | butyryl-CoA dehydrogenase | -2.3 | 4.72E-05 |
| ABUW_RS08050 | ABUW_1651 | hypothetical protein | -2.2 | 4.88E-04 |
| ABUW_RS11940 | ABUW_2454 | enoyl-CoA hydratase/isomerase family protein | -2.1 | 5.38E-05 |
| ABUW_RS00205 | ABUW_0042 | 2,5-didehydrogluconate reductase DkgB | -2.1 | 2.92E-04 |
| ABUW_RS18510 | ABUW_3803 | IS5-like element ISAba13 family transposase | -2.1 | 4.20E-02 |
| ABUW_RS13010 | ABUW_2679 | DUF4142 domain-containing protein | -2.1 | 1.66E-03 |
| ABUW_RS11945 | ABUW_2455 | acetyl/propionyl/methylcrotonyl-CoA carboxylase subunit alpha | -2.1 | 3.77E-05 |
| ABUW_RS18770 | ABUW_3855 | FMNH2-dependent alkanesulfonate monooxygenase | -2.0 | 4.20E-05 |
| ABUW_RS11935 | ABUW_2453 | methylcrotonoyl-CoA carboxylase | -2.0 | 3.77E-05 |
| ABUW_RS05000 | ABUW_1019 | sulfate ABC transporter permease subunit CysT | -2.0 | 2.92E-04 |
| ABUW_RS11950 | ABUW_2456 | hydroxymethylglutaryl-CoA lyase | -2.0 | 3.77E-05 |
| ABUW_RS10640 | ABUW_2189 | siderophore biosynthesis protein | -1.9 | 8.76E-05 |
| ABUW_RS11840 | ABUW_2433 | stress-induced protein | -1.9 | 1.83E-03 |
| ABUW_RS18365 | ABUW_3772 | acyl-CoA dehydrogenase | -1.9 | 6.15E-05 |
| ABUW_RS10160 | ABUW_2092 | 3-hydroxybutyrate dehydrogenase | -1.9 | 6.64E-05 |
| ABUW_RS13005 | ABUW_2678 | hypothetical protein | -1.9 | 6.16E-03 |
| ABUW_RS11930 | ABUW_2452 | isovaleryl-CoA dehydrogenase | -1.8 | 4.20E-05 |
| ABUW_RS12115 | ABUW_2489 | FMN-dependent NADH-azoreductase | -1.8 | 2.86E-04 |
| ABUW_RS10165 | ABUW_2093 | GntP family permease | -1.8 | 2.51E-05 |
| ABUW_RS10195 | ABUW_2099 | thiolase family protein | -1.8 | 3.22E-05 |
| ABUW_RS11575 | ABUW_2379 | taurine dioxygenase | -1.8 | 3.60E-04 |
| ABUW_RS10190 | ABUW_2098 | short-chain fatty acid transporter | -1.8 | 1.85E-05 |
| ABUW_RS11580 | ABUW_2380 | taurine ABC transporter permease TauC | -1.7 | 3.99E-05 |
| ABUW_RS10635 | ABUW_2188 | SidA/IucD/PvdA family monooxygenase | -1.7 | 3.25E-04 |
| ABUW_RS10005 | ABUW_2060 | hypothetical protein | -1.7 | 1.02E-02 |
| ABUW_RS09265 | ABUW_1903 | hypothetical protein | -1.7 | 1.81E-04 |
| ABUW_RS19395 | ABUW_4079 | hypothetical protein | -1.7 | 4.90E-02 |
| ABUW_RS18775 | ABUW_3856 | aliphatic sulfonate ABC transporter permease SsuC | -1.7 | 3.60E-04 |
| ABUW_RS04995 | ABUW_1018 | sulfate ABC transporter permease subunit CysW | -1.6 | 1.38E-04 |
| ABUW_RS12740 | ABUW_2626 | exo-alpha-sialidase | -1.6 | 2.18E-04 |
| ABUW_RS11915 | ABUW_2449 | class I SAM-dependent methyltransferase | -1.6 | 1.29E-04 |
| ABUW_RS11585 | ABUW_2381 | ATP-binding cassette domain-containing protein | -1.6 | 2.51E-05 |
| ABUW_RS11920 | ABUW_2450 | AMP-binding protein | -1.6 | 6.32E-05 |
| ABUW_RS05005 | ABUW_1020 | alpha/beta hydrolase | -1.6 | 5.58E-05 |
| ABUW_RS12605 | ABUW_2594 | glutathione-dependent formaldehyde dehydrogenase | -1.6 | 6.50E-04 |
| ABUW_RS11875 | ABUW_2440 | hypothetical protein | -1.6 | 1.71E-03 |
| ABUW_RS07145 | ABUW_1466 | DUF2171 domain-containing protein | -1.5 | 1.32E-03 |
| ABUW_RS09295 | ABUW_1909 | hypothetical protein | -1.5 | 6.88E-04 |
| ABUW_RS11605 | ABUW_2385 | LLM class flavin-dependent oxidoreductase | -1.5 | 6.15E-05 |
| ABUW_RS09260 | ABUW_1902 | sorbosone dehydrogenase family protein | -1.5 | 6.32E-05 |
| ABUW_RS11865 | ABUW_2438 | damage-inducible protein CinA | -1.5 | 3.94E-03 |
| ABUW_RS07690 | ABUW_1574 | AMP-binding protein | -1.5 | 6.72E-05 |
| ABUW_RS11365 | ABUW_2337 | LLM class flavin-dependent oxidoreductase | -1.5 | 1.16E-04 |
| ABUW_RS18420 | ABUW_3783 | CoA-acylating methylmalonate-semialdehyde dehydrogenase | -1.5 | 1.47E-04 |
| ABUW_RS07005 | ABUW_1438 | acyltransferase | -1.4 | 3.57E-03 |
| ABUW_RS05010 | ABUW_1021 | sulfate ABC transporter substrate-binding protein | -1.4 | 1.32E-03 |
| ABUW_RS07170 | ABUW_1471 | NirD/YgiW/YdeI family stress tolerance protein | -1.4 | 1.27E-03 |
| ABUW_RS05675 | ABUW_1159 | TetR/AcrR family transcriptional regulator | -1.4 | 2.29E-03 |
| ABUW_RS13040 | ABUW_2686 | hypothetical protein | -1.4 | 4.83E-03 |
| ABUW_RS13585 | ABUW_2797 | hypothetical protein | -1.4 | 1.54E-02 |
| ABUW_RS10630 | ABUW_2187 | DHA2 family efflux MFS transporter permease subunit | -1.4 | 9.98E-04 |
| ABUW_RS07820 | ABUW_1601 | hypothetical protein | -1.4 | 3.69E-02 |
| ABUW_RS00600 | ABUW_0121 | SH3 domain-containing protein | -1.4 | 1.04E-02 |
| ABUW_RS11925 | ABUW_2451 | TetR/AcrR family transcriptional regulator | -1.4 | 1.20E-04 |
| ABUW_RS18370 | ABUW_3773 | fatty acyl-AMP ligase | -1.4 | 1.91E-04 |
| ABUW_RS18355 | ABUW_3770 | non-ribosomal peptide synthetase | -1.4 | 1.33E-04 |
| ABUW_RS10025 | ABUW_2064 | hypothetical protein | -1.4 | 1.83E-03 |
| ABUW_RS19190 | ABUW_4033 | hypothetical protein | -1.4 | 1.32E-03 |
| ABUW_RS11845 | ABUW_2434 | hypothetical protein | -1.3 | 2.49E-03 |
| ABUW_RS18415 | ABUW_3782 | 3-hydroxyisobutyrate dehydrogenase | -1.3 | 9.52E-04 |
| ABUW_RS12420 | ABUW_2557 | epoxyqueuosine reductase QueH | -1.3 | 1.84E-04 |
| ABUW_RS12660 | ABUW_2606 | LamB/YcsF family protein | -1.3 | 1.21E-03 |
| ABUW_RS11750 | ABUW_2415 | serine acetyltransferase | -1.3 | 1.28E-03 |
| ABUW_RS15475 | ABUW_3186 | hypothetical protein | -1.3 | 2.24E-03 |
| ABUW_RS11755 | ABUW_2416 | hypothetical protein | -1.3 | 1.21E-03 |
| ABUW_RS11775 | ABUW_2420 | transporter substrate-binding domain-containing protein | -1.3 | 4.79E-05 |
| ABUW_RS11780 | ABUW_2421 | transporter substrate-binding domain-containing protein | -1.3 | 5.75E-04 |
| ABUW_RS00200 | ABUW_0041 | MFS transporter | -1.3 | 1.68E-04 |
| ABUW_RS18780 | ABUW_3857 | ATP-binding cassette domain-containing protein | -1.3 | 2.86E-04 |
| ABUW_RS10625 | ABUW_2186 | siderophore achromobactin biosynthesis protein AcsC | -1.3 | 1.11E-03 |
| ABUW_RS11270 | ABUW_2317 | hypothetical protein | -1.3 | 1.32E-03 |
| ABUW_RS19405 | ABUW_4082 | hypothetical protein | -1.3 | 2.45E-02 |
| ABUW_RS12220 | ABUW_2511 | hypothetical protein | -1.3 | 2.74E-02 |
| ABUW_RS11870 | ABUW_2439 | hypothetical protein | -1.3 | 2.30E-03 |
| ABUW_RS11360 | ABUW_2336 | SfnB family sulfur acquisition oxidoreductase | -1.2 | 1.81E-04 |
| ABUW_RS11860 | ABUW_2437 | iron-containing redox enzyme family protein | -1.2 | 2.30E-03 |
| ABUW_RS13435 | ABUW_2767 | NAD(P)/FAD-dependent oxidoreductase | -1.2 | 1.32E-03 |
| ABUW_RS10620 | ABUW_2185 | IucA/IucC family siderophore biosynthesis protein | -1.2 | 2.25E-03 |
| ABUW_RS15520 | ABUW_3197 | methionine synthase | -1.2 | 1.44E-03 |
| ABUW_RS12655 | ABUW_2605 | putative hydro-lyase | -1.2 | 1.38E-03 |
| ABUW_RS16140 | ABUW_3322 | hypothetical protein | -1.2 | 3.36E-04 |
| ABUW_RS16155 | ABUW_3325 | heavy metal translocating P-type ATPase | -1.2 | 1.28E-03 |
| ABUW_RS01375 | ABUW_0280 | sulfate ABC transporter substrate-binding protein | -1.2 | 1.16E-03 |
| ABUW_RS12215 | ABUW_2510 | hypothetical protein | -1.2 | 1.82E-02 |
| ABUW_RS09210 | ABUW_1891 | type 1 glutamine amidotransferase domain-containing protein | -1.2 | 9.72E-04 |
| ABUW_RS05720 | ABUW_1170 | acinetobactin non-ribosomal peptide synthetase subunit BasB | -1.2 | 3.36E-04 |
| ABUW_RS12665 | ABUW_2607 | divalent metal cation transporter | -1.2 | 6.71E-04 |
| ABUW_RS12725 | ABUW_2621 | hypothetical protein | -1.2 | 1.30E-03 |
| ABUW_RS08390 | ABUW_1723 | hypothetical protein | -1.2 | 6.52E-03 |
| ABUW_RS12885 | ABUW_2655 | hypothetical protein | -1.2 | 1.27E-03 |
| ABUW_RS00270 | ABUW_0055 | PQQ-dependent sugar dehydrogenase | -1.2 | 3.50E-04 |
| ABUW_RS12650 | ABUW_2604 | 5-oxoprolinase/urea amidolyase family protein | -1.1 | 9.23E-04 |
| ABUW_RS18765 | ABUW_3854 | sulfonate ABC transporter substrate-binding protein | -1.1 | 1.77E-03 |
| ABUW_RS11885 | ABUW_2442 | hypothetical protein | -1.1 | 1.53E-03 |
| ABUW_RS11785 | ABUW_2422 | amino acid ABC transporter ATP-binding protein | -1.1 | 5.06E-04 |
| ABUW_RS11620 | ABUW_2389 | cytochrome ubiquinol oxidase subunit I | -1.1 | 4.01E-04 |
| ABUW_RS11760 | ABUW_2417 | cysteine desulfurase | -1.1 | 2.66E-03 |
| ABUW_RS11745 | ABUW_2414 | rhodanese-like domain-containing protein | -1.1 | 4.43E-03 |
| ABUW_RS13035 | ABUW_2685 | hypothetical protein | -1.1 | 7.62E-03 |
| ABUW_RS17400 | ABUW_3575 | serine hydrolase family protein | -1.1 | 1.91E-03 |
| ABUW_RS07950 | ABUW_1629 | hypothetical protein | -1.1 | 2.16E-02 |
| ABUW_RS11240 | ABUW_2311 | fimbria/pilus periplasmic chaperone | -1.1 | 6.93E-04 |
| ABUW_RS12745 | ABUW_2627 | DUF2147 domain-containing protein | -1.1 | 6.78E-04 |
| ABUW_RS07830 | ABUW_1603 | GNAT family N-acetyltransferase | -1.1 | 3.93E-04 |
| ABUW_RS11910 | ABUW_2448 | hypothetical protein | -1.1 | 1.66E-03 |
| ABUW_RS12645 | ABUW_2603 | ATP-grasp domain-containing protein | -1.1 | 2.04E-03 |
| ABUW_RS07150 | ABUW_1467 | hypothetical protein | -1.1 | 2.24E-03 |
| ABUW_RS10350 | ABUW_2130 | alpha-ketoacid dehydrogenase subunit beta | -1.1 | 1.34E-03 |
| ABUW_RS11855 | ABUW_2436 | catalase HPII | -1.1 | 2.22E-03 |
| ABUW_RS11625 | ABUW_2390 | cytochrome d ubiquinol oxidase subunit II | -1.0 | 1.28E-03 |
| ABUW_RS10345 | ABUW_2129 | 2-oxo acid dehydrogenase subunit E2 | -1.0 | 1.02E-03 |
| ABUW_RS06275 | ABUW_1287 | hypothetical protein | -1.0 | 8.77E-03 |
| ABUW_RS05710 | ABUW_1168 | acinetobactin utilization protein BauF | -1.0 | 2.52E-03 |
| ABUW_RS13120 | ABUW_2703 | four-helix bundle copper-binding protein | -1.0 | 7.38E-03 |
| ABUW_RS16135 | ABUW_3321 | copper resistance system multicopper oxidase | -1.0 | 2.99E-04 |
| ABUW_RS15160 | ABUW_3122 | trehalose-phosphatase | -1.0 | 4.31E-02 |
| ABUW_RS05995 | ABUW_1227 | acyl-CoA dehydrogenase | -1.0 | 1.21E-03 |
| ABUW_RS18350 | ABUW_3769 | outer membrane lipoprotein-sorting protein | -1.0 | 4.33E-04 |
| ABUW_RS13775 | ABUW_2834 | hypothetical protein | -1.0 | 4.71E-03 |
| ABUW_RS05680 | ABUW_1161 | hypothetical protein | -1.0 | 7.38E-03 |

**Table S5: RNA-Seq expression versus qPCR expression in the AB00324 *gshA* mutant strain compared to the *A. baumanii* AB5075-UW wildtype strain.** Fold-changes of expression in RNA-Seq values represented as log_2_FC while qPCR values are represented as fold change with the maximum and minimal differences from the mean value in parentheses. Statistical analysis of qPCR data was done using Mann-Whitney. P-value represented with asterisks with * = *p*-value < 0.05.

| ***A. baumannii*** | **RNASeq Log_2_FC** | **qPCR Fold-Change *(gyrB)*** | **qPCR Fold-Change (16S)** |
| --- | --- | --- | --- |
| *gshA* | N/A | 1.1 (+0.2 -0.3) | 1.0 (+0.2 -0.3) |
| *gshB* | N/A | 1.0 (+0.1 -0.1) | 0.9 (+0.1 -0.1) |
| ABUW_1481 | 2.4 | 2.9 (+0.3 -0.2)* | 2.6 (+0.3 -0.2)* |
| ABUW_1477 | 3.2 | 7.1 (+1.9 -1.3)* | 6.3 (+1.6 -1.2)* |
| ABUW_2535 | 1.9 | 2.8 (+0.1 -0.1)* | 2.5 (+0.1 -0.2)* |
| ABUW_1572 | -2.6 | 0.3 (+0.0 -0.0)* | 0.2 (+0.0 -0.0)* |
| ABUW_2316 | -2.6 | 0.3 (+0.1 -0.1)* | 0.3 (+0.0 -0.1)* |
| ABUW_2096 | -3.2 | 0.5 (+0.2 -0.2)* | 0.5 (+0.1 -0.2)* |

**Table S6: Differential expression of genes involved in protection against oxidative stress in the AB00324 *gshA* mutant strain compared to the *A. baumanii* AB5075-UW wildtype strain.**

| **Annotation** | **Gene Name** | **Description** | **Log_2_FC** |
| --- | --- | --- | --- |
| ABUW_2436 | *katE* | Catalase | -1.1 |
| ABUW_3469 | *katG* | Catalase peroxidase | NDR |
| ABUW_2504 |  | Catalase family Peroxidase | -0.7 |
| ABUW_0628 |  | Peroxidase | -0.7 |
| ABUW_1216 |  | Superoxide dismutase | NDR |
| ABUW_0339 |  | Superoxide dismutase family protein | -0.6 |
| ABUW_2212 |  | Dyp-type peroxidase | NDR |
| ABUW_3745 |  | Dyp-type peroxidase | NDR |
| ABUW_2719 | *ahpC* | Peroxiredoxin | NDR |
| ABUW_0628 |  | Putative peroxiredoxin | -0.7 |
| ABUW_2294 |  | OsmC-like peroxiredoxin | NDR |
| ABUW_2740 |  | OsmC-like peroxiredoxin | NDR |
| ABUW_3027 |  | OsmC-like peroxiredoxin | NDR |
| ABUW_3750 |  | OsmC-like peroxiredoxin | NDR |
| ABUW_2355 |  | Glutathione peroxidase | -0.5 |
| ABUW_3729 |  | Glutathione peroxidase | -0.4 |
| ABUW_3264 | *ohrR* | Transcription factor | NDR |
| ABUW_3265 | *ohr* | OsmC-like peroxiredoxin | NDR |
